# Senescence in Cancer: Hallmarks, Paradoxes, and Therapeutic Promise

**DOI:** 10.1016/j.cell.2026.03.005

**Published:** 2026-04-03

**Authors:** Clemens Hinterleitner, Hailey V. Goldberg, Domhnall McHugh, Valentin J. A. Barthet, Aveline Filliol, Scott W. Lowe

**Affiliations:** 1 Department of Cancer Biology and Genetics, Memorial Sloan Kettering Cancer Center, New York, NY 10065, USA; 2 Weill Cornell Graduate School of Medical Sciences, New York, New York 10065, USA; 3 Howard Hughes Medical Institute, Chevy Chase, MD 20815, USA

## Abstract

Cellular senescence is a conserved stress-responsive program defined by durable proliferative arrest and extensive remodeling of chromatin, metabolism, intercellular signaling, and immune interactions. Initially described as a barrier to unlimited cell division, senescence is now recognized as a pleiotropic and heterogeneous biological process with roles in development, tissue repair, immune surveillance, tumor suppression, aging, fibrosis, and cancer progression. Despite its broad relevance, senescence remains challenging to define operationally, as its molecular features, functional outputs, and physiological consequences vary across cell types, tissues, and stimuli. This review summarizes core hallmarks of senescence while synthesizing how these features are differentially engaged, diversified, and repurposed across biological contexts. Focusing on cancer, we discuss how senescence influences tumor initiation, evolution, and therapeutic response through both cell-intrinsic and microenvironmental mechanisms. We further evaluate emerging strategies to therapeutically modulate senescence, highlighting both opportunities and unresolved challenges for precision intervention.

## I Introduction

Cellular senescence was first described over six decades ago by Hayflick and Moorhead as the process associated with replicative exhaustion of cultured fibroblasts^1^. In contrast to tumor-derived cell lines or virally transformed cells, which can proliferate indefinitely, normal fibroblasts possess an intrinsic limit to proliferation, entering a stable and irreversible arrest after an initial phase of exponential growth. What initially seemed like an arcane curiosity eventually captured the attention of biologists, who debated whether this proliferative barrier represented an evolved defense against tumorigenesis or instead reflected a ‘mitotic clock’ underlying organismal aging^2^. In hindsight, this early paradox foreshadowed a central theme that continues to define the field: the same biological program can apparently have beneficial and detrimental effects on tissues, depending on context and time.

Subsequent work established senescence as a stress-responsive cell fate triggered by a broad range of cellular and tissue insults, including telomere attrition, oncogenic signaling, or genotoxic stress^3,4^. Genetic studies in mice later demonstrated that cells bearing features of senescence are active drivers of tissue biology, suppressing tumorigenesis through durable cell-cycle arrest and immune-mediated clearance, or promoting fibrosis, chronic inflammation, and tissue dysfunction when they persist^3-10^. Together, these discoveries transformed senescence from a cell-culture curiosity into an organizing concept in biology – revealing both its therapeutic promise and profound conceptual challenges – and set the stage for efforts to understand, and ultimately control, this complex cellular state.

Early mechanistic studies firmly linked senescence to both cancer suppression and aging. Viral oncoproteins such as SV40 large T antigen, which disable the p53 and RB tumor suppressors, were shown to bypass replicative senescence, implicating senescence as a barrier to oncogenic transformation^11^. Subsequent work revealed the basis for this arrest: replicative senescence arises from progressive telomere shortening with serial cell division, a process prevented by telomerase, the enzyme that maintains telomere length and, remarkably, is reactivated in most cancers^12,13^. In contrast, telomerase inactivation in mice eventually leads to telomere attrition, tissue atrophy, and premature aging^14^, implicitly linking senescence to organismal aging. Together, these observations revealed a shared molecular logic: pathways that restrain malignant proliferation also impose limits on tissue renewal over time. The discovery that oncogenic RAS triggers a senescent-like arrest in early-passage fibroblasts extended this framework, demonstrating that senescence is not merely a passive consequence of proliferative exhaustion but an actively engaged, inducible response to deregulated oncogenic signaling^15^. This insight explained the co-requirement for an “immortalizing” oncogene in RAS-induced transformation of primary cells and established senescence as a regulated tumor-suppressive program ^16^.

Beyond growth arrest, senescence is distinguished by profound changes in how the senescent cell communicates with its tissue environment. Early studies showing that cultured senescent fibroblasts secrete matrix metalloproteinases and other extracellular factors^17-20^, suggesting that senescent cells might actively remodel their surroundings. These and related observations ultimately led to the conceptualization of the “senescence-associated secretory phenotype (SASP)”, a coordinated program of proteases, growth factors, and immune modulatory cytokines with the capacity to remodel and modulate immune survelliance^21-23^. Through this capacity for tissue communication, senescence acquires dual and often opposing biological roles: in tumor suppressive settings, SASP components can influence the immune mediated surveillance and eventual clearance of senescent cells, whereas in aged or chronically injured tissues, persistent (and perhaps altered) SASP activity can promote inflammation, fibrosis, and immune suppression^24-27^. This duality has positioned senescence as a canonical example of antagonistic pleiotropy, a theory describing trade-offs in which biological programs that enhance fitness and reproductive success early in life ultimately drive tissue decline and pathology with age^5^.

Despite its potential importance, historical nuances in how senescence has been defined and detected have hindered broader acceptance of the program’s relevance across the biomedical research community. Although senescence is well-characterized *in vitro*, its manifestations *in vivo* are heterogeneous and lack definitive markers that reliably distinguish senescence from related or overlapping cell states. Consequently, pinpointing senescent cells in tissues, and assigning them casual roles in physiology or disease, remains challenging. Equally unresolved is the paradox of “beneficial” versus “detrimental” senescence: why apparently similar programs can promote repair and regeneration in one setting yet drive inflammation and degeneration in another.

In this review, written in recognition of Hanahan and Weinberg’s seminal synthesis of the *Hallmarks of Cancer* ^28^, we argue that senescence can be understood through a similar lens: as a constellation of defining features that together shape cellular and tissue behavior in a deeply context-dependent manner. Rather than advancing a fixed operational definition as attempted elsewhere^29,30^, we frame senescence as a dynamic biological process whose features are variably engaged and intersect with related stress-adaptive cell states. We highlight emerging advances that reshape how senescence is defined and understood and consider how a more rigorous mechanistic framework may enable its rational therapeutic manipulation. We begin by outlining the molecular and phenotypic hallmarks that define the senescent state, emphasizing both their explanatory power and the challenges inherent in their interpretation *in vivo*.

## II. Senescence and the Hallmarks of Cancer

Despite the ongoing controversies surrounding its definition, measurement, and context-dependent outputs, cellular senescence increasingly emerges as a biological program positioned at the intersection of tissue injury, chronic inflammation, and tumorigenesis. Many of the pathways engaged during senescence mirror those activated during normal tissue repair, yet unlike acute wound-healing, cells with senescence-associated programs often persist, creating an unresolved inflammation that can profoundly shape tissue behavior. As cancers are often viewed as “wounds that do not heal” ^31^, it is then perhaps not surprising that senescence programs influence in some way each of Hanahan and Weinberg’s initial six cancer hallmarks.

In early neoplasia, oncogene-induced senescence (OIS) functions as a potent cell-intrinsic barrier to malignant transformation, directly opposing cancer-associated programs linked to uncontrolled proliferation, including “self-sufficiency in growth signals”, “limitless replicative potential”, and “insensitivity to antigrowth signals” ^32^. By enforcing durable arrest, OIS restrains the expansion of oncogene-expressing cells at a stage when transformation would otherwise be favored. In contrast, senescent cells that persist in developing tumor cells or their surrounding stroma engage pro-survival programs that support “evasion of apoptosis”.

The paradoxical nature of senescence is most apparent in processes governing “sustained angiogenesis” and “invasion and metastasis”. Through the SASP, senescent cells actively remodel their environment, and SASP components such as VEGF and matrix metalloproteinases produced by senescent stromal cells can fuel “sustained angiogenesis: and “invasion and metastasis” ^27,33-35^. Collectively, senescence-related programs mirror normal wound-healing responses, but when chronically engaged, become maladaptive – driving fibrosis, immune dysregulation, and tumor progression rather than repair. In this light, senescence can be viewed as a persistent, inflammation-linked repair program whose consequences depend on context, timing, and tissue composition.

The diverse output and complexity of senescence programs underscore the need for a framework that captures both the defining features of senescent cells and the diversity of their outputs in vivo. We therefore next consider the “*Hallmarks of Senescence*”: the molecular and phenotypic traits that characterize this state, as well as the challenges inherent in applying these criteria across tissues, disease states, and experimental systems.

## III. Hallmarks of senescence

**Figure 1:**
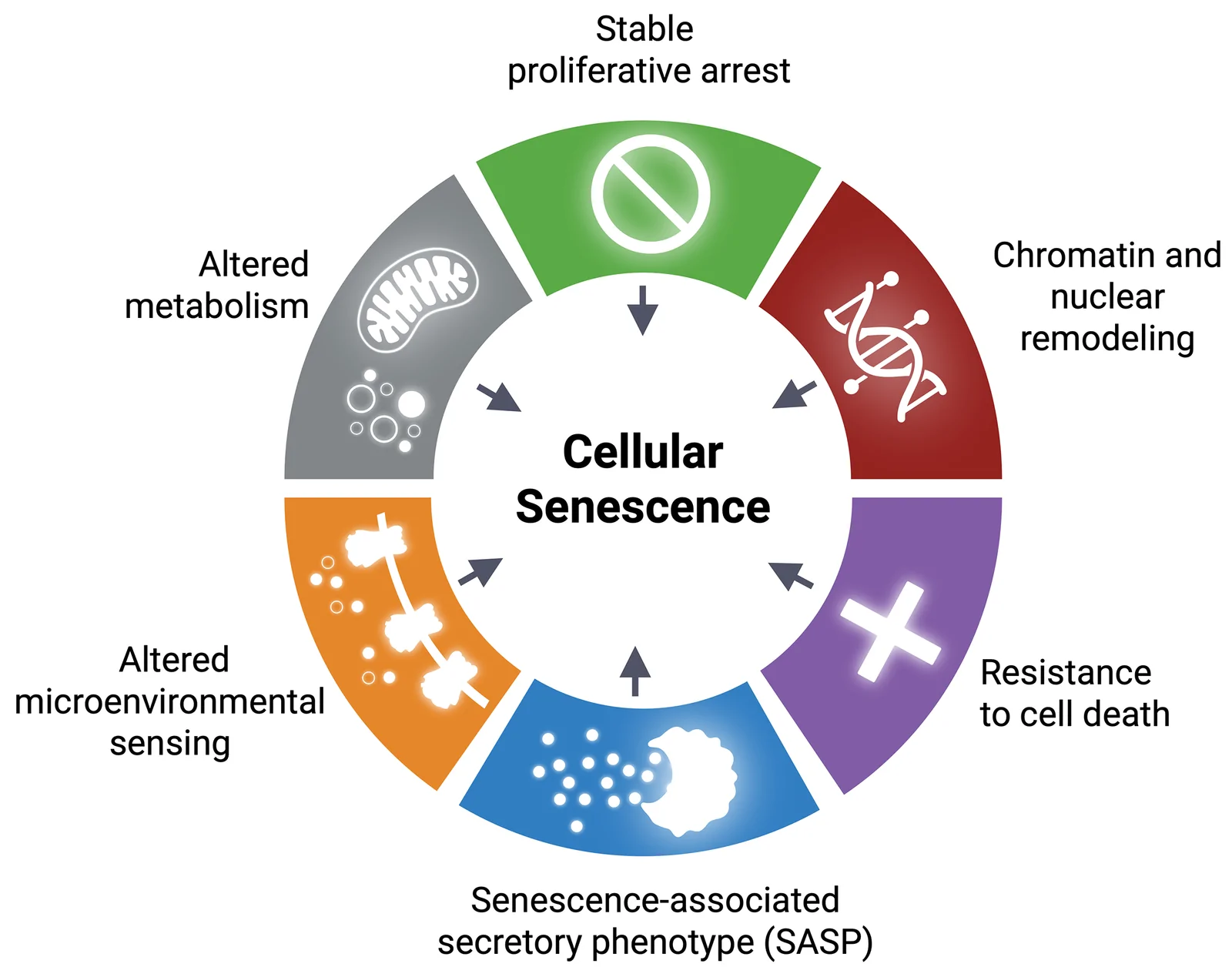
Hallmarks of Cellular Senescence Analogous to the “Hallmarks of Cancer,” the hallmarks of senescence summarize the most reproducible and biologically informative features of this complex, context-dependent state. Here we propose six interconnected dimensions to define senescence. First, a stable proliferative arrest, enforced by the p53/p21 and p16^INK4a-RB^ pathways, distinguishing senescence from reversible states. Second, chromatin and nuclear remodeling stabilize cell-cycle arrest while enabling senescence-associated transcriptional programs. Third, senescent cells acquire resistance to cell death, permitting persistence and creating therapeutic vulnerabilities. Fourth, the senescence-associated secretory phenotype (SASP), responsible for driving intercellular communication, driving immune surveillance, tissue repair, or chronic pathology. Fifth, remodeling of the cell-surface proteome changing microenvironmental sensing and immune recognition. Finally, extensive metabolic and lysosomal adaptations supporting survival, SASP production, and stress resistance, while also contributing to proteostatic and redox imbalance. Figure created using BioRender.

As the “*Hallmarks of Cancer*” framework provides a conceptual map for tumor biology^28^, the “*Hallmarks of Senescence*” outlined below are intended to capture the most reproducible and biologically informative features of this complex, context-dependent state. Derived primarily from studies in cultured fibroblasts, these hallmarks encompass six broad dimensions: (1) stable proliferative arrest, (2) chromatin and nuclear remodeling, (3) resistance to cell death, (4) the senescence-associated secretory phenotype, (5) altered microenvironmental sensing, and (6) altered metabolism (Figure 1).

**Stable proliferative arrest** is a defining feature of senescence and is mediated by the p53/p21 and p16^INK4a^/RB tumor-suppressor pathways^11,15,36^. These pathways converge to suppress E2F-driven transcription, including the cyclin A and E genes, thereby silencing genes required for DNA replication and cell-cycle progression^37,38^. This durable arrest is stabilized by a more repressive chromatin architecture at proliferation-associated loci, distinguishing senescence from quiescent states in which arrest is reversible and chromatin remains comparatively plastic^38-40^. Whereas functional studies demonstrate clear cooperation between p21 and p16 in reinforcing senescence in vitro^36,39,41^, recent single-cell transcriptomic analyses across senescence contexts in vivo suggest that these pathways can also operate independently, with p21-positive and p16-positive cells occupying distinct trajectory states^42^.

**Chromatin and nuclear remodeling** represent a second hallmark of senescence^38,43-49^. These changes include formation of senescence-associated heterochromatin foci (SAHF), loss of lamina-associated domains (LADs), and extensive enhancer rewiring^37,50-52,53^. At the level of histone regulation, senescence is accompanied by focal deposition of repressive marks at proliferation-associated loci (e.g., H3K9me3 and Polycomb-linked H3K27me3, with enrichment of histone variants such as macroH2A)^38,44,45^, coupled to activation of inflammatory and stress-responsive enhancers marked by H3K27ac and H3K4me1/3 and read by the bromodomain and extra-terminal domain (BET) protein BRD4^54^. Together, this chromatin-scale reorganization stabilizes proliferative arrest while simultaneously creating a permissive landscape for activation of senescence-associated transcriptional programs, including the SASP^54-56^.

Senescent cells also exhibit **resistance to cell death**, rendering them less susceptible to elimination in response to diverse stressors. This phenotype is achieved in part through induction of anti-apoptotic proteins (including BCL-2, BCL-XL and BCL-W), together with chromatin changes that suppress expression of pro-apoptotic genes^57-60^. Beyond apoptosis, senescent cells can acquire resistance to alternative cell-death modalities, including ferroptosis, consistent with their altered redox homeostasis and lipid metabolic state^61-63^. Resistance to cell death enables senescent-cell persistence, amplifying pathological outputs when senescence clearance fails. At the same time, their reliance on these survival programs creates context-dependent vulnerabilities that have been exploited to selectively target senescent cells^60^.

A fourth hallmark, the **senescence-associated secretory phenotype** (SASP), reflects a broad reprogramming of intercellular communication potential. Senescent cells secrete a pleiotropic mixture of cytokines, chemokines, MMPs, ECM components, growth factors, lipids, and extracellular vesicles that influence neighboring cell states^17,21,22,64^. Through the SASP, senescent cells can promote immune-mediated clearance of damaged or premalignant cells or, when persistent, drive chronic inflammation, fibrosis, and tumor progression^10,17,65,66^. The breath, plasticity, and capacity of SASP to propagate senescence or remodel tissue architecture distinguishes senescence from most other arrested states.

**Altered microenvironmental sensing** represents another defining feature of senescent cells that reflects broad remodeling of the cell-surface proteome, including changes in proteins governing adhesion, ligand recognition, and signal transduction. Senescence is associated with upregulation of surface receptors such as Notch, the IFN-γ receptor (IFNGR), uPAR, and the glycolipid GD3, with the precise repertoire varying by cell type, senescence trigger, and temporal context^67-74^. These alterations, often driven by NF-κB and/or interferon signaling, reshape how senescent cells respond to extracellular cues^75,76^, establishing feed-forward circuits that modulate SASP output and sustain inflammatory or pro-fibrotic states. In parallel, changes in NK-cell ligands and MHC-I expression shape immune recognition, influencing whether senescent cells are cleared or persist within tissues^68,71,77-79^. In therapy-induced senescence (TIS), enhanced antigen presentation through MHC I upregulation can engage CD4^+^ or CD8^+^ T cells, promoting anti-tumor immunity^71^.

Finally, ‘**altered metabolism’** emerges as a sixth hallmark of senescence. Senescent cells acquire marked changes in mitochondrial and lysosomal numbers, morphology and function, display altered redox balance, rewired glycolytic/OXPHOS states, increased dependence on metabolites such as NAD^+^ and a-ketoglutarate, and broad changes in lipid metabolism^80,81,82^. Such metabolic adaptations support growth arrest, apoptosis resistance, and SASP production^83-86^. Senescent cells also exhibit altered autophagy and marked expansion of the lysosomal compartment, leading to accumulation of senescence-associated β-galactosidase (SA-β-gal) activity, a widely used though imperfect senescence marker^87-89^. Collectively, these metabolic and lysosomal changes promote survival under stress while contributing to oxidative imbalance and disrupted proteostasis^80,90-94^.

Despite their utility, these hallmarks are neither universal nor exclusive to senescent states. SA-β-gal activity reflects lysosomal content and autophagic flux that varies between cell types independently of senescence^29,95,96^; p21^CIP1/WAF1^ is induced during DNA-damage responses that trigger reversible checkpoint arrest^97^; and SASP-like programs are activated in diverse cell types during infection and tissue injury, not all of which would be classified as senescent^98,99^. Classical senescence biomarkers are also detected upon extended quiescence or in certain quiescent tumor-cell states^100,101^. Moreover, p16^INK4a^ can be highly expressed in cancer cells lacking RB and/or p53^102^, where its induction reflects compensatory but futile pathway activation rather than durable proliferative arrest. Consistent with this, in breast ductal carcinoma *in situ*, lesions with high p16^high^/Ki67^low^ staining (senescence-like) carry a substantially lower risk of progression than those with p16^high^/Ki67^high^ staining^103 104^. Even structural features such as SAHF vary widely across cell types and species^105,106^. Collectively, these observations argue against fixed minimal or exclusionary criteria and instead support viewing senescence as a continuum of context-dependent, stress-adaptive states.

Large-scale transcriptomic analyses further support this view. The widely used SenMayo signature^107^, compiled from hundreds of datasets, shows strongest overlap with “epithelial-to-mesenchymal transition (EMT)”, a cell state classically linked to development and metastasis. Similarly, analysis of the Tabula Sapiens atlas^108^ highlights “monocytes” and “macrophages” as the cell types most strongly aligned with senescence-linked transcriptional programs, consistent with the long-recognized expression of canonical senescence markers (e.g. p16^INK4a^, SA-β-gal) in macrophage subsets^109-111^. This convergence has fueled debate over whether certain macrophage states are genuinely “senescent” or whether these widely used markers lack selectivity. In practice, both interpretations may apply: some macrophage populations may occupy “*senescence-like states*,” engaging features of the classical senescence program without necessarily satisfying all defining criteria. Recognizing this continuum shifts emphasis from rigid definitions to underlying biology, enabling more nuanced interpretation of damage-responsive, stress-adapted, and immune-modulatory states across tissues.

More broadly, the difficulty in defining senescence reflects a conceptual inversion in how the field has evolved. Rather than emerging from tissue- or organismal-level observations, the defining features of senescence were derived from fibroblast models *in vitro*, with *in vivo* biology subsequently retrofitted to these criteria, often incompletely. Because senescent cells actively rewire environmental sensing and cell-cell communication networks, the program that unfolds in tissues is inherently dynamic, heterogeneous, and shaped by lineage, microenvironment, and immune pressure—features not captured in reductionist cell culture systems. Single-cell and spatial profiling now reveal that “senescence” comprises a spectrum of related states (“senotypes”) defined by combinatorial molecular programs, functional behaviors, and tissue context rather than a fixed collection of markers^42^ – likely explaining its divergent roles in tissue repair, fibrosis, inflammation, and tumorigenesis^112,113^. Bridging gaps between culture models and tissues, and redefining senescence through its in vivo manifestations, will be essential for establishing hallmarks that accurately reflect its biological scope in cancer and beyond^29,30^. In this light, the *Hallmarks of Senescence* should be viewed as an evolving framework, analogous to the iterative refinement of the *Hallmarks of Cancer* as new biological insights and technologies have reshaped our understanding of tumor biology^114,115 116^.

## IV. Senescence mechanisms

**Figure 2:**
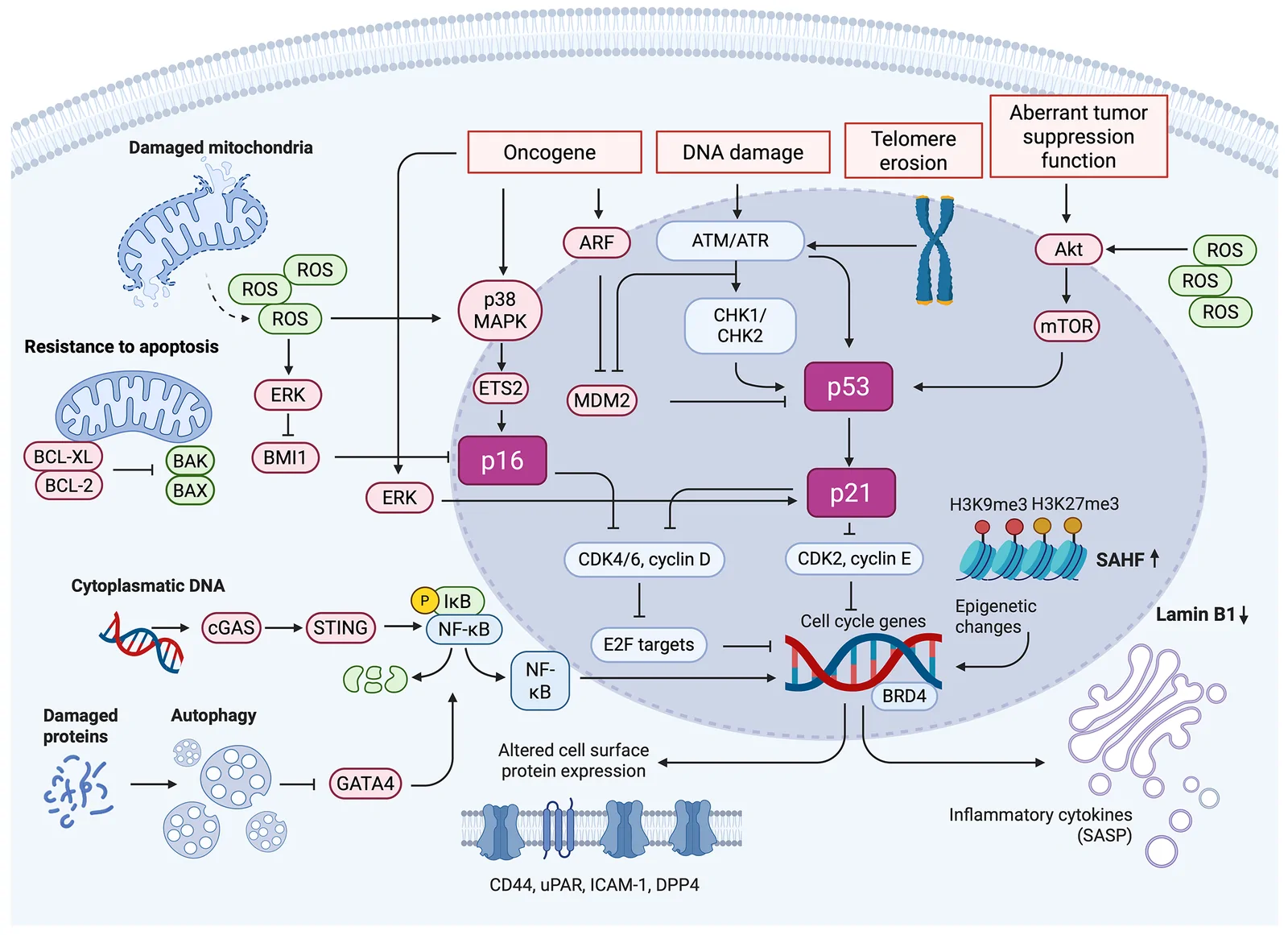
Cellular programs underlying senescence. Cellular senescence, triggered by oncogenes, DNA damage, telomere shortening, among others, is orchestrated by a transcriptional network centered on the p53–p21 and p16^INK4a/RB^ pathways, which converge to stably repress cyclins, CDKs, and E2F-dependent cell-cycle genes. Unlike quiescent cells, senescent cells are refractory to mitogenic signals due to a repressive chromatin landscape at proliferation-associated loci, characterized by Lamin B1 loss, SAHF formation, and large-scale three-dimensional genome reorganization. In parallel, senescence engages a distinct chromatin-accessible program that drives the senescence-associated secretory phenotype (SASP) and altered environmental sensing, mediated by regulators such as NF-κB and BRD4. This output includes inflammatory cytokines, growth factors, matrix-remodeling enzymes, and extensive remodeling of the cell-surface proteome, reshaping immune and stromal interactions. While cell-cycle arrest and tissue remodeling can intersect through shared chromatin mechanisms, SASP can also be regulated independently via autophagy and cytosolic DNA. Driven by genetic and metabolic imbalance senescent cells further confer apoptosis resistance through upregulation of BCL-2 family proteins. Figure created using BioRender.

At its core, senescence involves a coordinated transcriptional program that enforces durable proliferative arrest through the p53-p21 and p16-RB tumor-suppressor pathways^38,39^(Figure 2). *CDNK1A* (p21), a direct transcriptional target of p53, inhibits cyclin E/A–CDK2 complexes, whereas p16, encoded by the *CDKN2A* locus, inhibits cyclin D-dependent CDK4/6, with both pathways converging on RB to E2F-driven transcription of cell cycle genes^36,117,118^. p53 stabilization can occur via ATM/ATR–CHK1/2-dependent DNA damage response signaling^119-121,107,108^ or by oncogene-mediated triggering of ARF^15,122-125^, an alternative reading frame product of the *CDKN2A* locus. Both processes antagonize MDM2, a protein that facilitates p53 degradation^126^. By contrast, p16 induction occurs in part via chromatin remodeling and depression of the *CDKN2A* locus, involving H3K27 demethylation and coordinated actions of chromatin modifiers, lncRNAs, and remodeling complexes^127-133^. The frequent inactivation of *TP53*, *CDKN2A*, and *RB1* across human cancers underscores the importance of this core senescence module as an intrinsic barrier to tumorigenesis.

Unlike quiescent cells, senescent cells fail to respond to mitogenic signals, remain refractory to growth-factor stimulation and are unable to re-engage transcriptional programs required for proliferation^134^. This distinction reflects the establishment of a more repressive chromatin environment at E2F target genes, whereas these genes appear poised for reactivation in quiescence^38,39,135,136^. Key factors leading to this repressive state include downregulation of Lamin B1, formation of SAHF, and extensive three-dimensional genome reorganization that repositions DNA-replication and cell-cycle genes into transcriptionally inert nuclear compartments^137,138^. The resulting heterochromatin landscape is actively maintained by RB and chromatin-modifying complexes, including Suv39H1^139^, H3K9me and HP1^47^. Although RB is just one member of a family of functionally related proteins, including p107 and p130, it is uniquely responsible for this repressive chromatin architecture^39^. Notably, studies on OIS indicate that the conversion from a reversible arrest to a fully senescent state requires ongoing chromatin remodeling, imposing a temporal barrier before senescence becomes irreversible^140^.

In contrast to the repressive chromatin architecture sustaining proliferative arrest, the SASP and environmental-sensing modules arise from broad increases in chromatin accessibility that enable activation of gene transcription^49,55^. Induction of these loci requires an interplay between chromatin regulators such as BRD4, p300 and transcriptional regulators such as NF-κB, a master regulator of inflammation, as well as C/EBPβ, GATA4, and others, deployed in a gene- and context-dependent manner^22,54,56,141,142^.

**Figure 3:**
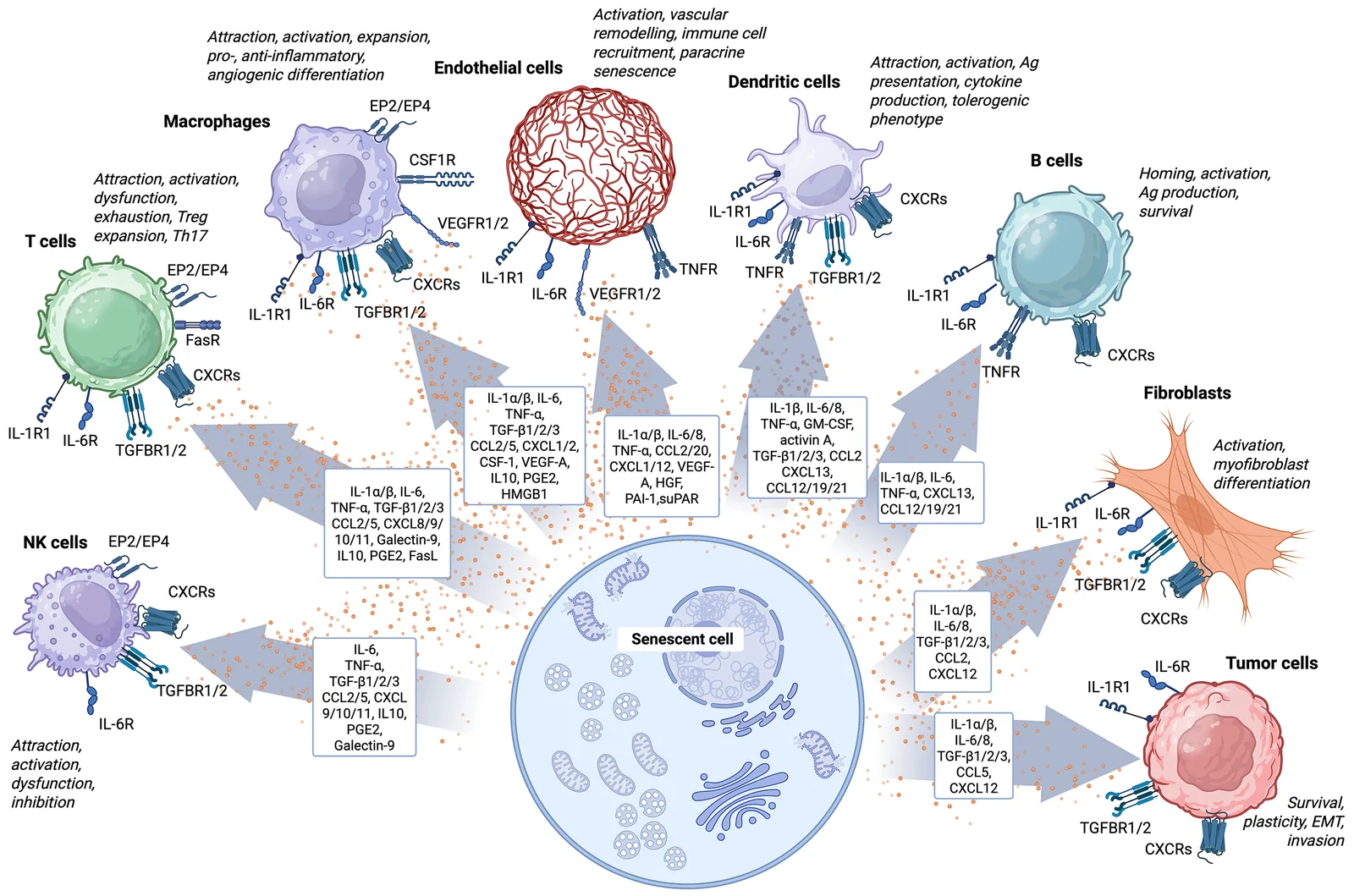
Intercellular communication networks in senescence Senescent cells coordinate extensive paracrine and juxtacrine signaling through the senescence-associated secretory phenotype (SASP), profoundly reshaping local tissue and immune landscapes. SASP factors, including inflammatory cytokines (e.g., IL-1α/b, IL-6, IL-8, TNF-α, TGFβ), chemokines (e.g., CCL2, CCL5, CXCL1/2), and growth factors (e.g., VEGF, HGF), modulate fibroblast and endothelial cell behavior and drive stromal and vascular remodeling. SASP components recruit NK cells to mediate senescent cell clearance, direct cytotoxic or regulatory responses in T cells, influence antibody production and activity in B cells, modulate antigen presentation by dendritic cells, and recruit and polarize macrophages toward pro- or anti-inflammatory states. Collectively, these pathways highlight senescent cells as central signaling hubs, integrating environmental sensing, extracellular matrix remodeling, and immune regulation. Figure created using BioRender.

The known activities of genes upregulated as part of the SASP and the reciprocal environmental-sensing program provide clues into how senescent cells might influence and respond to their surroundings^27,143,144^ (Figure 3). Senescent cells secrete pro-inflammatory cytokines (e.g. IL1α, IL6, IL8), chemokines (e.g. CCL2, CXCL1/2), growth factors (e.g. VEGF, HGF, PDGF), and matrix-remodeling enzymes (e.g. MMP1, MMP3, MMP13), as well as fibrinolytic regulators such as SERPINE1 and SERPINB2^27,64^. These factors collectively recruit and polarize immune cells remodel the extracellular matrix and reshape stromal behavior. In parallel, the cell-surface proteome is extensively altered: senescent cells upregulate adhesion molecules (e.g. ICAM1, VCAM1, CD44), anchored cell-surface and transmembrane proteins (e.g., uPAR, DPP4), and a spectrum of immune modulator proteins, including immune evasion factors (e.g. PD-L1, PD-L2, CD47) or NK-cell ligands (e.g. MICA/B, ULBPs). Additional changes in cytokine or growth factor receptors (e.g., IL1R, IL6R, TGFβR, IFNGR, CSFR1) have the potential to establish autocrine and paracrine feedback loops that reinforce senescence identity and/or shape immune interactions^34,64,70,145-149^.

Whether the two most prominent arms of senescence – durable proliferative arrest and tissue remodeling via SASP – are mechanistically inseparable or operate as semi-independent programs remains unresolved. Accumulating evidence supports a modular view in which these outputs are coordinated but not obligatorily coupled. One intersection point involves polycomb-mediated chromatin regulation, as loss of H3K27 methylation can derepress *CDKN2A* alongside subsets of SASP genes, linking stable arrest with tissue remodeling^150^. However, several SASP regulators function independently of cell-cycle control: mTOR signaling regulates translation of MAPKAPK2 and membrane-bound IL-1α to shape inflammatory SASP outputs^151,152^, while p38MAPK sustains mTOR and NF-κB activity downstream of senescence-inducing stress^153^. In addition, innate immune sensing of cytosolic nucleic acids can amplify SASP programs independently of canonical arrest pathways^76^. From an evolutionary perspective, coupling cell-cycle arrest to SASP may coordinate tumor suppression with microenvironmental alert, whereas partial independence provides flexibility across contexts, allowing inflammatory programs to be deployed without durable arrest and vice versa^154^.

Despite their stable proliferative arrest, senescent cells remain metabolically active, though rewired in a manner the durability of arrest and the magnitude and composition of the SASP^155, 155^. Across senescence triggers, mitochondrial remodeling is prominent, characterized by increased mitochondrial mass but impaired respiratory efficiency, altered TCA flux, defective mitophagy, and elevated mitochondrial ROS, which sustain DNA damage signaling and reinforces stable arrest^155^. Concomitant perturbations in redox homeostasis and NAD^+^ metabolism link mitochondrial stress to AMPK–p53 signaling and epigenetic control of inflammatory gene programs, exemplified by mitochondrial dysfunction–associated senescence (MiDAS)^83^, highlighting mitochondria as active regulators rather than passive casualties of senescence. In parallel, rewiring of nutrient utilization, including increased glycolytic flux, lipid remodeling, and altered lysosomal biogenesis and autophagy, supports the energetic and biosynthetic demands of chronic cytokine and extracellular matrix production, collectively coupling metabolic state to persistent arrest, SASP programming, tissue remodeling, and immune modulation^93,155,156^

Despite this framework, senescence-related programs are heterogeneous and highly context dependent. Major drivers of heterogeneity are epigenetic variation, which shapes chromatin accessibility and transcriptional output, and the SASP, which comprises hundreds of secreted factors whose combinatorial output can yield inflammatory, immunosuppressive, fibrogenic, or fibrolytic environments depending on lineage and tissue context^27^. In tumors, genetic background provides another source of heterogeneity: for example, TIS can be triggered in tumor cells harboring inactivating mutations in canonical senescence regulators (*TP53*, *RB1*, and/or *CDKN2A*), likely yielding molecularly distinct outcomes that differ from normal cells. Indeed, partial engagement of senescent programs may generate dormant, therapy-tolerant states capable of cell cycle reentry and contributing to tumor relapse. Defining the molecular determinants that specify these distinct senescence-associated states, and how they influence tissue remodeling, fibrosis, and cancer progression, remains a central challenge for the field.

## V. Tumor suppression

Because the durable proliferative arrest enforced by senescence is fundamentally incompatible with uncontrolled cell division, senescence has long been viewed as a cell intrinsic barrier to tumorigenesis. Evidence supporting this role in human cancer is largely correlative but compelling: inactivating mutations in canonical senescence regulators such as *TP53*, *CDKN2A*, and *RB1*, together with activating *TERT* promoter mutations, rank among the most frequent genetic events across malignancies^126,157-159^. Their prevalence implies that most human cancers must evade senescence at some point during their evolution.

Direct experimental evidence that senescence actively suppresses tumorigenesis derives from studies of OIS. OIS was first observed in primary human fibroblasts following enforced oncogenic RAS expression, leading to a stable growth arrest accompanied by p53 and p16^INK4a 15^ through engagement of MAPK pathway and other stress-activated signaling cascades^160-163^. Beyond RAS, other oncogenic stimuli – such as oncogenic BRAF, cyclin E overexpression, or PTEN loss–can also provoke senescence^121,160,164-167^. Landmark studies in the mid-2000s demonstrated that cells with senescent features accumulate in premalignant lesions such as melanocytic nevi, and that genetic disruption of senescence cooperates with oncogenic drivers to accelerate tumorigenesis in vivo^15,160,164,165,168-171^. Evidence that OIS limits tumorigenesis in humans is also correlative, but the frequent co-occurrence of *KRAS* and *TP53* mutations in human tumors provides provocative supports for its functional relevance^157,172^.

Importantly, oncogenic signaling does not uniformly induce senescence but instead does so when a threshold is breached. Early studies involving high level RAS expression reliably induced senescence, whereas models expressing lower levels did not^173^. Subsequent in vivo studies clarified this discrepancy: in lung carcinogenesis, low-level oncogenic signaling promotes benign adenoma formation, whereas malignant progression required higher KRAS output and selection for cells that bypass senescence^174,175^. Similarly, in the liver, varying NRAS signaling intensities produces distinct outcomes, with high-level activation inducing senescence and blocking tumorigenesis, and intermediate signaling promotes tumor formation with distinct histopathological features^176^. The nature of the molecular sensors that detect such thresholds remains a mystery.

**Figure 4:**
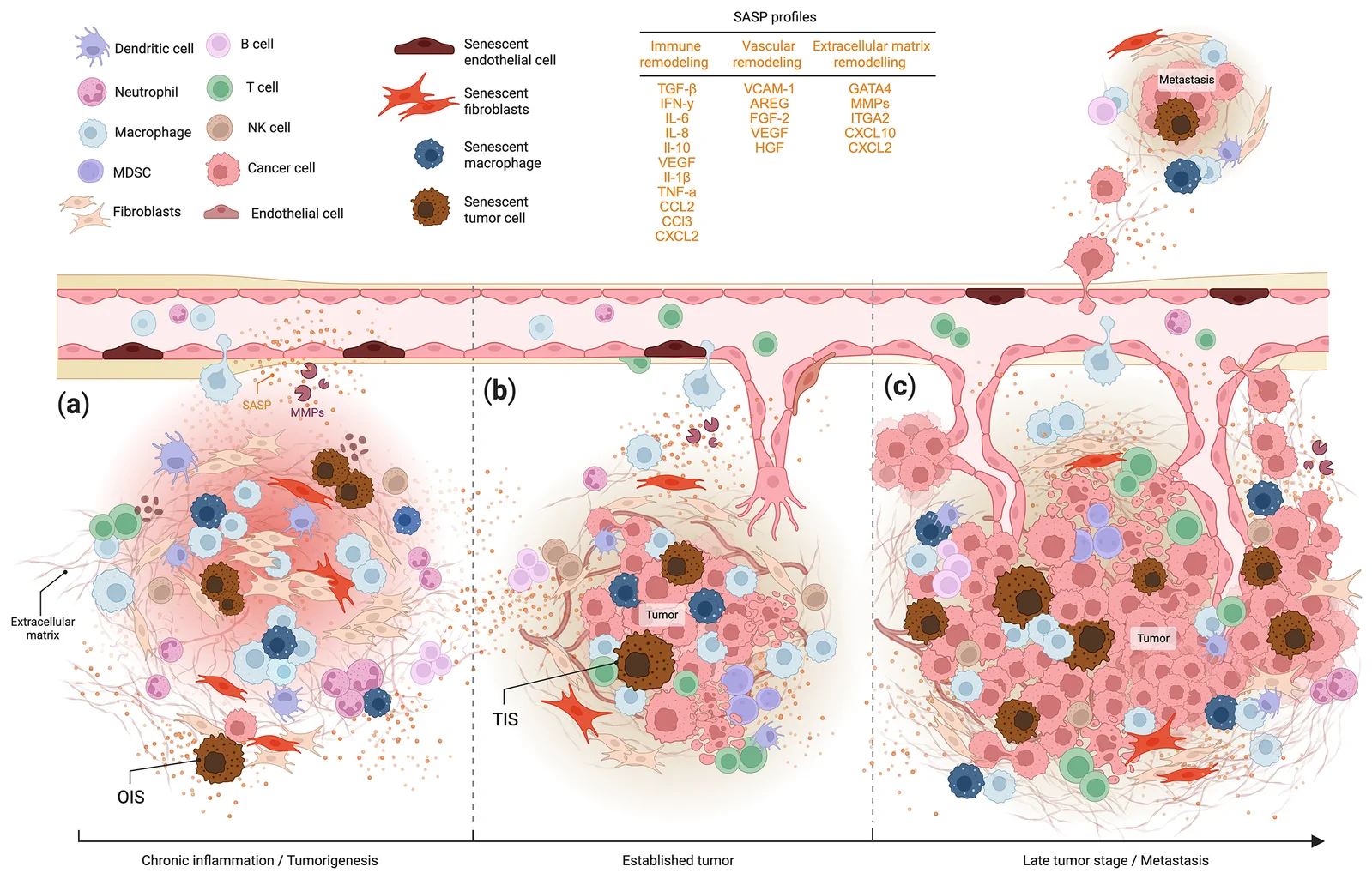
Spatial and temporal evolution of senescence in the TME Senescence arises dynamically in multiple cell types during tumor development, displaying distinct temporal and contextual features. (**a**) In chronic inflammation and premalignant tissues, oncogene-induced senescence (OIS) emerges in epithelial cells, acting as a barrier to malignant transformation. Concurrently, tissue damage and inflammation induce senescent or senescent-like states in fibroblasts, endothelial cells, and macrophages, modulating the local microenvironment to either restrain or promote tumor progression. (**b**) In established tumors, therapy-induced senescence (TIS) occurs in tumor cells and stromal populations, driving SASP-mediated extracellular matrix remodeling, vascular modulation, and recruitment and polarization of immune cells, including dendritic cells, NK cells, T cells, and macrophages. (**c**) In late-stage tumors and metastatic niches, persistent senescence in fibroblasts, endothelial cells, and macrophages sustains chronic SASP activity, promoting fibrosis, immune suppression, and pro-metastatic remodeling of the tumor microenvironment. Figure created using BioRender.

The advent of single-cell and spatial transcriptomic technologies has enabled the direct identification of senescent-like cells within premalignant lesions in vivo. These analyses reveal discrete cellular states that arise during early neoplasia and function to restrain malignant progression (Figure 4). In pancreatic cancer, for example, cells exhibiting senescence-like features emerge during the transition from benign precursor lesions to invasive carcinoma^177^. In liver cancer, the transcriptional identity of these states depends on the magnitude of oncogenic RAS signaling and correlates with molecular features of advanced disease^176^. Collectively, these studies indicate that senescence programs are engaged in cells undergoing epigenetic and transcriptional plasticity en route to malignant fates^178^. Such senescent-like states, marked by p53 activation, p16^INK4a^ induction, and altered cell–cell communication networks, constitute a barrier to malignant transformation that is breached only when tumor-suppressive networks fail (Figure 4). This role is in line with earlier functional studies showing that senescence, p53, p16, and indeed senescence constrain epigenetic reprogramming during induced pluripotency^179,180^, underscoring a broader function for senescence as a barrier to cell plasticity.

Beyond its cell-intrinsic arrest, immune surveillance is a critical component of senescence-mediated tumor suppression. Early evidence came from a study exploring the role of p53 loss in tumor maintenance in mice, where p53 reactivation in established tumors triggered senescence and tumor regression, a process that was dependent on immune clearance rather than cell intrinsic apoptosis^9^. Subsequent work demonstrated that both innate and adaptive populations can help eliminate senescent cells during tumor initiation and therapy response^8,181,182,10^. Mechanistically, SASP programs governed by NF-κB and BRD4 promote immune recruitment and activation^54,78,141^, while type I and II interferon signaling enhances antigen presentation and MHC-I expression, facilitating immune recognition^68,71^. These pathways constitute one of the most conserved transcriptional signatures across senescent states^154,183,184^. Notably, genes encoding type I IFNs are adjacent to *CDKN2A* on chromosome 9p21 and are frequently co-deleted in human cancers, and their combined inactivation accelerates KRAS-driven tumorigenesis by promoting immune evasion^185^.

Mechanistic insights into senescence surveillance have emerged primarily from models in which senescence is reinstated in established tumors, revealing a coordinated interplay between innate and adaptive immunity in a context-dependent manner^9,34,68,186^. In some contexts, macrophages phagocytose senescent cells^187^ and are recruited in part via p21-dependent CXCL14 secretion^188^ while, in other settings, p21 activity in macrophages promotes inflammatory phenotypes that attract CD8^+^ cytotoxic T cells^97^. NK cells recognize senescent cells through increased NKG2D–ligand interactions (e.g. MICA, ULBP2)^8,9,181,182^, while dendritic cells facilitate antigen presentation, and neutrophils may act as early sensors of oncogenic stress^189^. Importantly, this immune response is not uniformly tumor suppressive: senescent cells can also recruit immunosuppressive myeloid populations^190^ and inhibit NK-cell surveillance^148^.

Adaptive immunity also contributes to senescence surveillance through SASP-driven remodeling of the cell-surface proteome, enhancing antigen presentation and immune recognition^191^. One mechanism involves upregulation of IFN-γ receptors and attenuation of negative IFN regulators, amplifying MHC-I expression and CD8^+^ T-cell–mediated clearance^68^. Consistent with this, senescent cells elicit robust antitumor immunity in vaccination models^71,77,192^, responses dependent on both dendritic cells and cytotoxic T cells. Whether similar adaptive immune responses eliminate senescent cells during premalignancy, and the nature of the neoantigens involved, remain open questions, as do the determinants dictating engagement of innate versus adaptive immunity.

Although malignant progression typically requires the acquisition of mutations that bypass senescence-associated proliferative arrest, vestiges of the chromatin remodeling and the SASP programs often persist in advanced cancers^193,194^. In fact, analysis of the three-dimensional chromatin organization of tumor cells indicate that they aquire features with aged or senescent cells rather than their precancerous counterparts^193,195,196^. As a result, certain cytotoxic or targeted cancer therapies can reengage senescence programs, even in tumors with compromised p53 or p16 pathways^80,120,193,195-197^. In some settings, this TIS is sufficient to trigger immune surveillance and subsequent tumor regression, providing an explanation for why some cytostatic agents can promote tumor regressions in patients.

In summary, senescence suppresses tumorigenesis through durable proliferative arrest, tissue remodeling, and immune surveillance. While repression of this program enables malignant progression, residual or inducible senescent-like states create therapeutic opportunities. Nonetheless, senescence is not uniformly tumor suppressive, emphasizing the need for a nuanced understanding of how this complex process can be harnessed safely and effectively in cancer therapy.

## VI. Physiological roles of senescence

The tumor-suppressive functions of senescence are somewhat paradoxical from an evolutionary perspective. If senescence evolved as a tumor-suppressive mechanism, how can this be reconciled with the fact that most human cancers arise after reproductive age, when selective pressure is diminished? One possibility is that senescence-related processes confer early-life benefits that, if absent, would enable cancer development before loss of reproductive fitness. Consistent with this idea, individuals with germline inactivating mutations in *TP53* (Li–Fraumeni syndrome), *RB1* (retinoblastoma), or *CDKN2A* (familial melanoma) – three core senescence regulators – frequently develop multiple malignancies early in life^198-200^. A second, non-mutually exclusive possibility is that senescence-related programs evolved to support normal physiological processes, with tumor suppression emerging as a secondary consequence or later adaptation. In this regard, studies linking senescence to embryonic development and wound healing suggest two plausible hypotheses.

Transient senescent-like cell populations appear in multiple embryonic structures, including the limb bud, mesonephros, and inner ear, where they contribute to tissue patterning and are subsequently cleared by macrophages^201 202^. Similar programs have been observed in zebrafish and naked mole rats, suggesting evolutionary conservation^203 204^. “Developmental senescence” differs from more canonical programs in that it occurs independently of classical DNA damage responses and instead relies on TGF-β– and p21-dependent signaling; notably, lineage-tracing studies indicate that some of these cells can re-enter the cell cycle^205^. Consistent with this distinction, mice harboring constitutive deletions of core senescence regulators (e.g., *Trp53* and *Cdkn2a*) are prone to malignant transformation yet exhibit relatively mild developmental phenotypes.

A potential physiological role for senescence that has proven more experimentally tractable has emerged from studies of wound healing and tissue repair. In multiple injury models, the induction of senescent-like states in the vasculature and stromal compartments, at least when present transiently, promotes tissue remodeling and regeneration^8,98,206^. For example, p53-dependent senescence in hepatic stellate cells facilities fibrosis resolution upon cessation of chronic chemical injury^8^, while p16-positive cells with senescence features promote wound repair in skin and lung^98,206^. p16-positive endothelial cells similarly contribute to hepatic vascular remodeling during liver regeneration^207,208^. These effects are mediated by SASP components such as EREG, PDGF-AA, HGF and several MMPs, and by signaling pathways including TGF-β and Notch, which coordinate communication among the fibroblast, immune, and epithelial compartments^23,72,73,209^. In some settings, senescent cells can even enhance regenerative plasticity in neighboring cells, linking senescence to tissue renewal and reprogramming^180,210^. From this perspective, senescence reflects a conserved damage-responsive and tissue-adaptive program that is repeatedly deployed during normal physiology, and whose misregulation, rather than mere presence, drives pathology.

As discussed below, senescence-related programs can be either beneficial or detrimental to wound repair, providing a useful framework for understanding how senescence contributes to a broader spectrum of pathological processes. Cancers, for example, co-opt features of wound-healing, angiogenesis, stromal remodeling, and immune modulation, and chronic activation of senescence-associated pathways may reinforce these malignant phenotypes^211^. Likewise, many degenerative diseases characterized by senescent-cell accumulation arise from chronic tissue damage, where repeated activation of reparative programs drives maladaptive tissue remodeling. Intriguingly, while enforcing EMT and stemness in some settings^212,213^, senescent cells may also suppress EMT in other contexts and limit the emergence of mesenchymal or stem-like populations with tumorigenic potential program^67,72^. While an attractive connection, many of the predictions based on these ideas have yet to be directly tested through systematic perturbation of defined senescent subpopulations in vivo.

Collectively, these observations indicate that senescence naturally functions as both a facilitator of regeneration and a barrier to transformation, with its biological impact dictated by context, timing, and persistence. A deeper understanding of these physiological roles will be essential to determine how perturbations of the senescence program contribute to tissue dysfunction, aging, and cancer; and to inform the development of therapeutic strategies that eliminate pathogenic senescent cells while preserving their beneficial roles in repair and homeostasis.

## VII. Deleterious effects of senescence

Despite the well-established roles of senescence in tumor suppression and tissue repair, it is now clear that the persistence of senescent or senescent-like cells can exert deleterious effects on tissue biology. Across both humans and experimental models, cells with senescence features accumulate with age and in settings of chromic tissue damage^5,214^. This accumulation is consistently associated with functional decline and a broad spectrum of age-related pathologies, including pulmonary fibrosis, osteoarthritis, metabolic liver disease, neurodegeneration, cardiovascular dysfunction, and adipose-tissue decline^65,215-219^. In these contexts, senescent cells are identified by shared molecular hallmarks (e.g. p16, p21, SA–β-gal, and SASP transcripts)^26^, and arise across diverse lineages, including fibroblasts, epithelial cells, macrophages, endothelial cells, adipocytes, and glia, with their prevalence and phenotypic features varying by tissue and disease context^65,215-219^. Although these observations are largely correlative, their consistency across tissues, organisms, and disease settings has raised the possibility that senescent cell accumulation is not merely a marker of tissue damage, but an active contributor to age-associated dysfunction.

Direct evidence that senescent cells drive age-related tissue decline has come from genetic models that enable their selective elimination based on expression of p16, one of the most well-validated senescence markers. The INK-ATTAC mouse^220^ expresses a drug-inducible FKBP-caspase-8 fusion protein under the control of the p16^INK4a^ promoter, allowing for selective ablation of p16-expressing cells upon treatment with the dimerizing agent AP20187. Clearance of accumulated p16-positive cells in both progeroid and naturally aged mice delays multiple aging phenotypes, including frailty, sarcopenia, cataract formation, cardiovascular dysfunction, and loss of hematopoietic regenerative capacity, while improving overall physiological function and extending lifespan^220,221^. These studies establish that p16-positive cells – and by association, senescent cells – are active contributors to organismal aging rather than passive bystanders, and that their removal can improve tissue function.

Subsequent reporter and effector strains incorporating luciferase, fluorescent tags, Cre recombinase and other suicide transgenes have expanded the ability to visualize, purify, and eliminate senescent subsets in vivo^98,220,222-226^. Using these tools, senescent-like cells have been implicated in disease settings beyond natural aging, including atherosclerosis ^218^, lung fibrosis^65^, osteoarthritis^215^, neurodegeneration^217^, and metabolic dysfunction^219^. Notably, these p16-positive senescent populations typically constitute only a small fraction of tissue-resident cells, often just a few percent, yet their elimination markedly improves organ function across models. This disproportionate impact likely reflects the paracrine reach of the SASP, which propagates inflammatory and profibrotic cues beyond the limited number of senescent cells present. Still, these studies have limitations: as mentioned previously, not all senescent cells express p16^42^ and not all p16-expressing cells are senescent^102,103^.

Most of the evidence linking senescent-like cells to human disease is correlative, though genome-wide association studies provide provocative hints of a causal role. Specifically, single-nucleotide polymorphisms on chromosome 9p21 occur near *CDKN2A* locus are strongly associated with a spectrum of age-related pathologies, including cardiovascular disease, type 2 diabetes, and other chronic disorders often linked to aberrant senescence programs^227,228^. While these associations do not establish senescence (or specifically *CDKN2A* expression) as the driving mechanism, they connect germline variation near a core senescence regulator to diseases in which senescent cells have been implicated experimentally.

Senescence also shapes cancer development and treatment responses in important ways. While senescence induction by cytotoxic therapies can contribute to treatment outcomes enforcing proliferative arrest or enhancing immune surveillance, the induction of TIS in normal tissues likely contributes to treatment-associated toxicities. Cytotoxic agents such as radiation, etoposide, and doxorubicin readily induce senescence in normal cells in culture^229-231^, and doxorubicin causes accumulation of senescent-like cells in tissues in a manner that is mitigated by ablation of p16-positive cells^35^. The resulting tissue dysfunction resembles age-associated decline and may underlie the “premature aging” phenotypes observed in pediatric cancer survivors exposed to genotoxic therapies early in life^232^.

Beyond these systemic effects, senescent stromal cells can promote tumor initiation, progression, and metastasis through remodeling the tissue microenvironment. Early studies demonstrated that co-cultures of pre-malignant epithelial cells with senescent cancer-associated fibroblasts (CAFs) dramatically increase their ability to form tumors upon transplantation into mice, even when the senescent cells comprise a minority of the stromal compartment^17,21,22,231^. Senescent CAFs also mediate immune suppression and promote progression of breast, pancreatic and prostate cancer cells^233-235^ and drive more plastic, aggressive tumor cell state in lung cancer^236^. Chronic tissue injury and inflammation – which foster senescent cell accumulation – increase susceptibility to tumor initiation in organs such as the liver^237,238^. SASP components, including IL-6, IL-8, CXCL1/2, MMPs, and other growth and matrix-remodeling factors (e.g. cathepsins, serine proteases, ADAM/ADAMTS proteases and integrins) reinforce malignant phenotypes, promote EMT, stimulate invasion, and create permissive niches for metastatic dissemination^144,213,239,240^.

Taken together, these findings indicate that prolonged or dysregulated engagement of senescence programs can convert a protective cellular response into a driver of tissue dysfunction and tumor-promoting ecosystems. At the same time, the potency and malleability of these programs suggest that senescence is not merely a correlate of pathology, but a biological process whose modulation may be harnessed therapeutically, provided its diverse outputs can be selectively controlled.

## VIII. Harnessing Senescence Biology Therapeutically

Despite the challenges in precisely defining senescent states, it has become clear that senescence-associated biology is highly potent and contributes to a spectrum of human diseases. Although no FDA-approved therapies were developed with senescence as their explicit target, many widely used agents, including cytotoxic chemotherapies, radiation, CDK4/6 inhibitors, targeted kinase inhibitors, and BCL-2 family antagonists, robustly induce, reinforce, or modulate senescence programs, and a component of their clinical activity likely derives from these effects ^214,241^. The observations imply that senescence is pharmacologically actionable and can, in principle, be deliberately manipulated for therapeutic benefit.

Across diverse disease contexts, two broad and complementary therapeutic strategies have emerged. The first focuses on inhibiting or reprogramming the deleterious consequences of persistent senescent cells within aged or damaged tissues, where senescent cells sustain chronic inflammation, fibrosis, and immune suppression, and create a favorable environment for tumor initiation and progression. In cancer, such senescence-blunting strategies can also enable T cell penetration into tumors, providing avenues for improved immunotherapies. A second strategy aims to harness the tumor suppressive elements of senescence for anti-cancer therapy. Such approaches aim to improve tumor control by leveraging durable proliferative arrest and the capacity of senescent cells to stimulate immune surveillance. Most current therapeutic efforts fall within one, or attempt to integrate both, of these frameworks.

A major focus has been on the development of senolytics, agents designed to eliminate senescent cells by exploiting vulnerabilities intrinsic to senescent states^242,243^. Transcriptomic and functional screens revealed that senescent cells disproportionately rely on anti-apoptotic BCL2 family proteins, proteostasis networks, stress-kinase signaling, and ion transport proteins to resist cell death. These dependencies have translated into pharmacologic strategies: navitoclax, a BCL-2/BCL-X_L_ inhibitor, preferentially kills multiple senescent cell types^244^; HSP90, NMT and mTOR inhibitors exploit heightened proteotoxic stress^91,94,245^; cardiac glycosides perturb proton transport ^86,246^; and natural products like fisetin expand senolytic chemical space with improved tolerability^247,248^. Combination regimens, including the dasatinib–quercetin (D+Q), show senolytic activity across diverse tissues and disease models^249,250^, underscoring the breadth of pathways that can be leveraged to eliminate senescent cells.

In parallel, senomorphic strategies aim to suppress or reprogram senescence-associated outputs (e.g. SASP) without necessarily eliminating senescent cells. Bromodomain and extra-terminal domain (BET) protein inhibitors, for example, attenuate SASP transcription by disrupting BRD4-dependent enhancer activation^54^ and reduce fibrosis in preclinical models^251,252^, although dose-limiting toxicities have constrained their clinical utility^253-256^. JAK inhibitors provide a second senomorphic approach, dampening SASP-driven inflammation and fibrosis by interrupting IL-1–NF-κB–JAK/STAT signaling^257^. Although senomorphic agents do not remove senescent cells and may therefore yield less durable responses than senolytics^242^, there are pathological contexts in which transient suppression of inflammatory signaling may be preferable to wholesale clearance, particularly when senescent cell subsets retain essential roles in tissue integrity or repair.

Proof-of-principle studies demonstrate that senolytic or senomorphic interventions can restore tissue function and resilience across organ systems, including hematopoietic recovery after chemotherapy ^243^, metabolic improvement during aging ^219,258^, and vascular rejuvenation in atherosclerotic or aged mice ^259^. Intermittent D+Q therapy extends lifespan and improves physical performance in aged mice ^249^, and early clinical studies, including an open-label pilot in idiopathic pulmonary fibrosis, suggest feasibility and tolerability of such protocols^260^. While promising, many of these agents have pleiomorphic effects, raising the possibility that therapeutic benefit reflects mechanisms beyond senolysis alone. Nevertheless, these studies set the stage for the use of senolytics to treat age-related tissue decline or senescence-related pathologies.

Despite growing promise, major challenges remain. Many senolytics target pathways essential for normal cell survival, raising concerns about on-target toxicities, as, for instance, exemplified by navitoclax-induced thrombocytopenia due to megakaryocyte dependence on BCL-XL ^244^. Most were discovered empirically and have incompletely defined mechanisms of action, complicating prediction of off-target effects. Toxicities, including cytopenias, gastrointestinal disturbances, liver dysfunctions, and cardiovascular risk, may limit use in frail individuals or heavily pretreated cancer patients ^249,260^. Moreover, since not all senescent cell populations are deleterious, indiscriminate clearance risks impairing wound healing, liver regeneration, or tumor suppression ^10,207^. These challenges underscore the need for next-generation therapies with improved mechanistic precision and therapeutic windows.

Achieving such specificity is an active area of innovation. Galacto-conjugated prodrugs exploit SA-b-gal activity to restrict cytotoxic payloads to senescent cells^261^, while antibody and cell-based strategies exploit remodeling of the senescent cell surfaceome. For example, the urokinase plasminogen activator receptor (uPAR) is broadly upregulated across senescent states in fibrosis and other pathological contexts, including in “immune-cold” tumors with dense fibrotic stroma. In murine models, chimeric antigen receptor (CAR) T cells targeting uPAR selectively deplete senescent cells in vivo, remodel profibrotic stroma, and ameliorate age- and fibrosis-associated pathologies with remarkably limited toxicities ^70,262,263^. In solid tumor models, the effects of uPAR CAR T cells on the profibrotic and immune suppressive stroma enables better T cell penetration and potentiates their anti-tumor effects. Notably, CAR T cells directed against NK-cell ligands upregulated on senescent cells (e.g., NKG2D ligands) also display senolytic activity in aged mice and nonhuman primates^264^. Together, these findings support the broader concept that senescence-enriched surface proteins can be exploited for selective targeting and may provide a generalizable strategy to improving senolytic specificity.

In cancer therapy, interest in senescence-based interventions has grown with the recognition that many standard treatments engage senescence programs that contribute to therapeutic responses. Radiation and DNA-damaging chemotherapies induce durable growth arrest and, in some contexts, activate SASP-dependent immune surveillance^35,265^. Targeted therapies can similarly engage senescence-related processes^266,267^, including inhibitors of CDK4/6 inhibitors (which mimic p16^INK4a^) ^268^, EGFR ^269^, RAS/MAPK^270^. Because senescence induction is slow and multidimensional, conventional high-throughput assays optimized for rapid readouts often fail to capture it^271^, complicating drug discovery in this space.

These observations have motivated “one–two punch” strategies, in which senescence induction is followed by selective elimination of the resulting senescent population^7^. Since senescent cells display heightened dependence on anti-apoptotic and stress-survival pathways^243^, senescence induction creates therapeutic windows for senolytic targeting^80,246,261,271-273^. For example, CDK4/6-induced senescence sensitizes cancer cells to BCL-XL or BCL-2 antagonists, enhancing tumor control^274^, and combined functional genomics and kinase screening has identified CDC7 - mTOR inhibitor combinations as a novel senescence-modulating approach^275^. Nonetheless, TIS induction is rarely uniform, such that vulnerabilities confined to senescent subpopulations may allow non-senescent clones to persist and drive relapse. Optimizing the timing, magnitude, and uniformity of senescence induction will be essential for such combinations to be effective.

Beyond cytostasis, TIS can actively remodel the tumor–immune interface in therapeutically favorable ways. Senescent cells upregulate the antigen-presentation machinery, interferon-stimulated genes, stress ligands, cytokines, and chemokines that recruit NK cells, macrophages, dendritic cells, and T cells^10,187^. Importantly, immune-mediated clearance represents a natural senolytic mechanism: senescent cells are intrinsically poised for immune recognition and elimination, and TIS amplifies many of the surface and secreted cues that engage this process. Senescence-inducing treatments, including CDK4/6 inhibition, oncogene blockade, and targeted pathway inhibition, activate interferon signaling, enhance antigen presentation, and boost cytotoxic CD8^+^ T-cell and NK-cell activity^34,68,186,270^. These programs can synergize with immune checkpoint blockade, in part owing to upregulation of PD-L1/2 and SASP-mediated immune recruitment^78,276,277^. Although not designed with “one-two punch” approaches in mind, clinically-approved chemo-immunotherapy regiments, such as those used in NSCLC, combine ICB with cytotoxic chemotherapy backbones that are potent inducers of senescence^278,279^. Moreover, senescence-driven immune activation may support cross-recognition of non-senescent tumor cells, a concept supported by rare abscopal-like responses following radiation^280^, a canonical senescence inducer.

Taken together, current work highlights the therapeutic potential and unresolved complexity of targeting senescence in cancer and other senescence-associated pathologies. Realizing this potential will require deeper mechanistic insight to enable selective intervention with minimized on-target toxicity, together with robust biomarkers to establish whether clinical responses truly reflect engagement of senescence programs. Establishing such predictive principles is essential to move senescence-modulating therapies from empirically derived interventions toward precise, mechanism-informed strategies. If successful, this shift could not only expand immunotherapy responsiveness and reshape tumor ecosystems but also position senescence biology as a broadly actionable axis for therapeutic intervention across cancer and age-related disease.

## IX. Future Directions and Outstanding Questions

Over the past six decades, our understanding of cellular senescence has evolved from an in vitro curiosity into a complex, multidimensional biological program with broad physiological and pathological relevance. Senescence-related programs shape embryogenesis, coordinate wound repair, suppress tumorigenesis, and engage immune surveillance; yet senescent-like states can also promote chronic inflammation, organ fibrosis, aging, and, in cancer, foster tumor-promoting microenvironments. Reconciling these opposing roles will require deeper mechanistic insight and experimental strategies capable of capturing the full stimulus-, cell state-, and tissue-dependent complexity senescence in vivo.

A persistent challenge is the accurate identification of senescent cells in normal tissues and disease states. Although p16- and p21-based reporter and cell ablation mouse strains have propelled the field forward, reliance on single markers and static transcriptional signatures risks conflating senescence with other stress-associated programs, including DNA damage response, inflammation, EMT, and/or tumor cell dormancy^281,282^. Rather than fueling debates over nomenclature, these overlaps should be viewed as an opportunity to integrate insights across disciplines, revealing context-specific manifestations of shared stress-adaptive programs. Disentangling these relationships therefore demands a shift away from static definitions toward frameworks that conceptualize senescence as a dynamic, interactive process embedded within tissue ecosystems. Single-cell, spatial, and functional genomic approaches offer the resolution needed to understand how senescent states interface with surrounding stromal and immune compartments, how these interactions differ across developmental and disease states, and how age or injury alters the capacity for senescence recognition and clearance.

A second central challenge moving forward is to achieve a mechanistic understanding of the duality of senescence and how distinct senescent programs give rise to divergent biological outcomes. Although senescent cells can promote tissue repair and tumor suppression, their persistence or maladaptive deployment drives chronic inflammation, fibrosis, and therapy resistance^4,6,283^. Key unresolved questions therefore center on the molecular logic by which senescent states diversify: how SASP composition, chromatin state, metabolism, and immune engagement vary across senescent states; how these features shift with age, injury, or treatment; and why TIS is immunostimulatory in some contexts yet immunosuppressive in others. Recent cell-type specific ablation studies underscore this complexity: during chronic liver injury, p16^+^ endothelial cells can facilitate tissue repair, whereas p16^+^ macrophages exacerbate fibrosis, producing opposing outcomes within the same tissue^208^. Together, these observations emphasize that senescence is not a monolithic state to be named, but a biology to be decoded.

Finally, translating senescence biology into effective cancer therapies will require selective, durable, and well-tolerated strategies to induce, eliminate, or reprogram senescent states. Current approaches are largely empirical and often directed at broadly acting survival pathways, remain variably selective and frequently limited by toxicity – a major issue in frail or heavily pretreated cancer patients. Defining which senescent populations should be eliminated, preserved, or altered, and how these populations evolve during disease progression or treatment, remains a critical objective. In this context, surfaceome features, metabolic dependencies, and state-restricted vulnerabilities offer a rational framework for selectively targeting pathogenic senescent populations while sparing those with beneficial roles in tissue integrity and repair. As mechanistic understanding deepens, efforts to therapeutically modulate senescence are poised to follow earlier inflection points in precision oncology—shifting from empirically discovered, broadly acting interventions toward mechanism-informed strategies capable of reshaping tumor ecosystems, expanding immunotherapy responsiveness, and durably altering disease trajectories.

## References

[1] Hayflick L, and Moorhead PS. (1961). The serial cultivation of human diploid cell strains. Exp Cell Res 25, 585–621. 10.1016/0014-4827(61)90192-6. 13905658

[2] Hayflick L. (1965). THE LIMITED IN VITRO LIFETIME OF HUMAN DIPLOID CELL STRAINS. Exp Cell Res 37, 614–636. 10.1016/0014-4827(65)90211-9. 14315085

[3] Campisi J. (2005). Senescent cells, tumor suppression, and organismal aging: good citizens, bad neighbors. Cell 120, 513–522. 10.1016/j.cell.2005.02.003. 15734683

[4] Campisi J, and d'Adda di Fagagna F. (2007). Cellular senescence: when bad things happen to good cells. Nat Rev Mol Cell Biol 8, 729–740. 10.1038/nrm2233. 17667954

[5] Campisi J. (2013). Aging, cellular senescence, and cancer. Annu Rev Physiol 75, 685–705. 10.1146/annurev-physiol-030212-183653. 23140366 PMC4166529

[6] Muñoz-Espín D, and Serrano M. (2014). Cellular senescence: from physiology to pathology. Nat Rev Mol Cell Biol 15, 482–496. 10.1038/nrm3823. 24954210

[7] Wang L, Lankhorst L, and Bernards R. (2022). Exploiting senescence for the treatment of cancer. Nat Rev Cancer 22, 340–355. 10.1038/s41568-022-00450-9. 35241831

[8] Krizhanovsky V, Yon M, Dickins RA, Hearn S, Simon J, Miething C, Yee H, Zender L, and Lowe SW. (2008). Senescence of activated stellate cells limits liver fibrosis. Cell 134, 657–667. 10.1016/j.cell.2008.06.049. 18724938 PMC3073300

[9] Xue W, Zender L, Miething C, Dickins RA, Hernando E, Krizhanovsky V, Cordon-Cardo C, and Lowe SW. (2007). Senescence and tumour clearance is triggered by p53 restoration in murine liver carcinomas. Nature 445, 656–660. 10.1038/nature05529. 17251933 PMC4601097

[10] Kang TW, Yevsa T, Woller N, Hoenicke L, Wuestefeld T, Dauch D, Hohmeyer A, Gereke M, Rudalska R, Potapova A, (2011). Senescence surveillance of pre-malignant hepatocytes limits liver cancer development. Nature 479, 547–551. 10.1038/nature10599. 22080947

[11] Shay JW, West MD, and Wright WE. (1992). Re-expression of senescent markers in deinduced reversibly immortalized cells. Exp Gerontol 27, 477–492. 10.1016/0531-5565(92)90003-i. 1330670

[12] Harley CB, Futcher AB, and Greider CW. (1990). Telomeres shorten during ageing of human fibroblasts. Nature 345, 458–460. 10.1038/345458a0. 2342578

[13] Bodnar AG, Ouellette M, Frolkis M, Holt SE, Chiu CP, Morin GB, Harley CB, Shay JW, Lichtsteiner S, and Wright WE. (1998). Extension of life-span by introduction of telomerase into normal human cells. Science 279, 349–352. 10.1126/science.279.5349.349. 9454332

[14] Rudolph KL, Chang S, Lee HW, Blasco M, Gottlieb GJ, Greider C, and DePinho RA. (1999). Longevity, stress response, and cancer in aging telomerase-deficient mice. Cell 96, 701–712. 10.1016/s0092-8674(00)80580-2. 10089885

[15] Serrano M, Lin AW, McCurrach ME, Beach D, and Lowe SW. (1997). Oncogenic ras provokes premature cell senescence associated with accumulation of p53 and p16INK4a. Cell 88, 593–602. 10.1016/s0092-8674(00)81902-9. 9054499

[16] Collado M, Blasco MA, and Serrano M. (2007). Cellular senescence in cancer and aging. Cell 130, 223–233. 10.1016/j.cell.2007.07.003. 17662938

[17] Krtolica A, Parrinello S, Lockett S, Desprez PY, and Campisi J. (2001). Senescent fibroblasts promote epithelial cell growth and tumorigenesis: a link between cancer and aging. Proc Natl Acad Sci U S A 98, 12072–12077. 10.1073/pnas.211053698. 11593017 PMC59769

[18] Goldstein S, Moerman EJ, Jones RA, and Baxter RC. (1991). Insulin-like growth factor binding protein 3 accumulates to high levels in culture medium of senescent and quiescent human fibroblasts. Proc Natl Acad Sci U S A 88, 9680–9684. 10.1073/pnas.88.21.9680. 1719537 PMC52782

[19] West MD, Pereira-Smith OM, and Smith JR. (1989). Replicative senescence of human skin fibroblasts correlates with a loss of regulation and overexpression of collagenase activity. Exp Cell Res 184, 138–147. 10.1016/0014-4827(89)90372-8. 2551704

[20] Zeng G, and Millis AJ. (1996). Differential regulation of collagenase and stromelysin mRNA in late passage cultures of human fibroblasts. Exp Cell Res 222, 150–156. 10.1006/excr.1996.0019. 8549657

[21] Coppé JP, Patil CK, Rodier F, Sun Y, Muñoz DP, Goldstein J, Nelson PS, Desprez PY, and Campisi J. (2008). Senescence-associated secretory phenotypes reveal cell-nonautonomous functions of oncogenic RAS and the p53 tumor suppressor. PLoS Biol 6, 2853–2868. 10.1371/journal.pbio.0060301. 19053174 PMC2592359

[22] Kuilman T, Michaloglou C, Vredeveld LC, Douma S, van Doorn R, Desmet CJ, Aarden LA, Mooi WJ, and Peeper DS. (2008). Oncogene-induced senescence relayed by an interleukin-dependent inflammatory network. Cell 133, 1019–1031. 10.1016/j.cell.2008.03.039. 18555778

[23] Acosta JC, O'Loghlen A, Banito A, Guijarro MV, Augert A, Raguz S, Fumagalli M, Da Costa M, Brown C, Popov N, (2008). Chemokine signaling via the CXCR2 receptor reinforces senescence. Cell 133, 1006–1018. 10.1016/j.cell.2008.03.038. 18555777

[24] He S, and Sharpless NE. (2017). Senescence in Health and Disease. Cell 169, 1000–1011. 10.1016/j.cell.2017.05.015. 28575665 PMC5643029

[25] Childs BG, Gluscevic M, Baker DJ, Laberge RM, Marquess D, Dananberg J, and van Deursen JM. (2017). Senescent cells: an emerging target for diseases of ageing. Nat Rev Drug Discov 16, 718–735. 10.1038/nrd.2017.116. 28729727 PMC5942225

[26] Tchkonia T, Zhu Y, van Deursen J, Campisi J, and Kirkland JL. (2013). Cellular senescence and the senescent secretory phenotype: therapeutic opportunities. J Clin Invest 123, 966–972. 10.1172/jci64098. 23454759 PMC3582125

[27] Coppé JP, Desprez PY, Krtolica A, and Campisi J. (2010). The senescence-associated secretory phenotype: the dark side of tumor suppression. Annu Rev Pathol 5, 99–118. 10.1146/annurev-pathol-121808-102144. 20078217 PMC4166495

[28] Hanahan D, and Weinberg RA. (2000). The hallmarks of cancer. Cell 100, 57–70. 10.1016/s0092-8674(00)81683-9. 10647931

[29] Ogrodnik M, Carlos Acosta J, Adams PD, d'Adda di Fagagna F, Baker DJ, Bishop CL, Chandra T, Collado M, Gil J, Gorgoulis V, (2024). Guidelines for minimal information on cellular senescence experimentation in vivo. Cell 187, 4150–4175. 10.1016/j.cell.2024.05.059. 39121846 PMC11790242

[30] Suryadevara V, Hudgins AD, Rajesh A, Pappalardo A, Karpova A, Dey AK, Hertzel A, Agudelo A, Rocha A, Soygur B, (2024). SenNet recommendations for detecting senescent cells in different tissues. Nat Rev Mol Cell Biol 25, 1001–1023. 10.1038/s41580-024-00738-8. 38831121 PMC11578798

[31] Dvorak HF. (1986). Tumors: wounds that do not heal. Similarities between tumor stroma generation and wound healing. N Engl J Med 315, 1650–1659. 10.1056/nejm198612253152606. 3537791

[32] Braig M, and Schmitt CA. (2006). Oncogene-induced senescence: putting the brakes on tumor development. Cancer Res 66, 2881–2884. 10.1158/0008-5472.Can-05-4006. 16540631

[33] Coppe JP, Kauser K, Campisi J, and Beausejour CM. (2006). Secretion of vascular endothelial growth factor by primary human fibroblasts at senescence. J Biol Chem 281, 29568–29574. 10.1074/jbc.M603307200. 16880208

[34] Ruscetti M, Morris J.P.t., Mezzadra R, Russell J, Leibold J, Romesser PB, Simon J, Kulick A, Ho YJ, Fennell M, (2020). Senescence-Induced Vascular Remodeling Creates Therapeutic Vulnerabilities in Pancreas Cancer. Cell 181, 424–441.e421. 10.1016/j.cell.2020.03.008. 32234521 PMC7278897

[35] Demaria M, O'Leary MN, Chang J, Shao L, Liu S, Alimirah F, Koenig K, Le C, Mitin N, Deal AM, (2017). Cellular Senescence Promotes Adverse Effects of Chemotherapy and Cancer Relapse. Cancer Discov 7, 165–176. 10.1158/2159-8290.Cd-16-0241. 27979832 PMC5296251

[36] Beauséjour CM, Krtolica A, Galimi F, Narita M, Lowe SW, Yaswen P, and Campisi J. (2003). Reversal of human cellular senescence: roles of the p53 and p16 pathways. Embo j 22, 4212–4222. 10.1093/emboj/cdg417. 12912919 PMC175806

[37] Nijwening JH, Geutjes EJ, Bernards R, and Beijersbergen RL. (2011). The histone demethylase Jarid1b (Kdm5b) is a novel component of the Rb pathway and associates with E2f-target genes in MEFs during senescence. PLoS One 6, e25235. 10.1371/journal.pone.0025235. 21980403 PMC3181323

[38] Narita M, Nũnez S, Heard E, Narita M, Lin AW, Hearn SA, Spector DL, Hannon GJ, and Lowe SW. (2003). Rb-mediated heterochromatin formation and silencing of E2F target genes during cellular senescence. Cell 113, 703–716. 10.1016/s0092-8674(03)00401-x. 12809602

[39] Chicas A, Wang X, Zhang C, McCurrach M, Zhao Z, Mert O, Dickins RA, Narita M, Zhang M, and Lowe SW. (2010). Dissecting the unique role of the retinoblastoma tumor suppressor during cellular senescence. Cancer Cell 17, 376–387. 10.1016/j.ccr.2010.01.023. 20385362 PMC2889489

[40] Ye X, Zerlanko B, Kennedy A, Banumathy G, Zhang R, and Adams PD. (2007). Downregulation of Wnt signaling is a trigger for formation of facultative heterochromatin and onset of cell senescence in primary human cells. Mol Cell 27, 183–196. 10.1016/j.molcel.2007.05.034. 17643369 PMC2698096

[41] Schmitt CA, Fridman JS, Yang M, Lee S, Baranov E, Hoffman RM, and Lowe SW. (2002). A senescence program controlled by p53 and p16INK4a contributes to the outcome of cancer therapy. Cell 109, 335–346. 10.1016/s0092-8674(02)00734-1. 12015983

[42] Saul D, Jurk D, Doolittle ML, Kosinsky RL, Han Y, Zhang X, Franco AC, Kim SY, Wyles SP, Prakash YS, (2025). Distinct senotypes in p16- and p21-positive cells across human and mouse aging tissues. Embo j 44, 7295–7325. 10.1038/s44318-025-00601-2. 41162753 PMC12669595

[43] Narita M, Narita M, Krizhanovsky V, Nuñez S, Chicas A, Hearn SA, Myers MP, and Lowe SW. (2006). A novel role for high-mobility group a proteins in cellular senescence and heterochromatin formation. Cell 126, 503–514. 10.1016/j.cell.2006.05.052. 16901784

[44] Zhang R, Poustovoitov MV, Ye X, Santos HA, Chen W, Daganzo SM, Erzberger JP, Serebriiskii IG, Canutescu AA, Dunbrack RL, (2005). Formation of MacroH2A-containing senescence-associated heterochromatin foci and senescence driven by ASF1a and HIRA. Dev Cell 8, 19–30. 10.1016/j.devcel.2004.10.019. 15621527

[45] Chandra T, Kirschner K, Thuret JY, Pope BD, Ryba T, Newman S, Ahmed K, Samarajiwa SA, Salama R, Carroll T, (2012). Independence of repressive histone marks and chromatin compaction during senescent heterochromatic layer formation. Mol Cell 47, 203–214. 10.1016/j.molcel.2012.06.010. 22795131 PMC3701408

[46] Sati S, Bonev B, Szabo Q, Jost D, Bensadoun P, Serra F, Loubiere V, Papadopoulos GL, Rivera-Mulia JC, Fritsch L, (2020). 4D Genome Rewiring during Oncogene-Induced and Replicative Senescence. Mol Cell 78, 522–538.e529. 10.1016/j.molcel.2020.03.007. 32220303 PMC7208559

[47] Zhang R, Chen W, and Adams PD. (2007). Molecular dissection of formation of senescence-associated heterochromatin foci. Mol Cell Biol 27, 2343–2358. 10.1128/mcb.02019-06. 17242207 PMC1820509

[48] De Cecco M, Criscione SW, Peckham EJ, Hillenmeyer S, Hamm EA, Manivannan J, Peterson AL, Kreiling JA, Neretti N, and Sedivy JM. (2013). Genomes of replicatively senescent cells undergo global epigenetic changes leading to gene silencing and activation of transposable elements. Aging Cell 12, 247–256. 10.1111/acel.12047. 23360310 PMC3618682

[49] Swanson EC, Manning B, Zhang H, and Lawrence JB. (2013). Higher-order unfolding of satellite heterochromatin is a consistent and early event in cell senescence. J Cell Biol 203, 929–942. 10.1083/jcb.201306073. 24344186 PMC3871423

[50] Shimi T, Butin-Israeli V, Adam SA, Hamanaka RB, Goldman AE, Lucas CA, Shumaker DK, Kosak ST, Chandel NS, and Goldman RD. (2011). The role of nuclear lamin B1 in cell proliferation and senescence. Genes Dev 25, 2579–2593. 10.1101/gad.179515.111. 22155925 PMC3248680

[51] Sadaie M, Salama R, Carroll T, Tomimatsu K, Chandra T, Young AR, Narita M, Pérez-Mancera PA, Bennett DC, Chong H, (2013). Redistribution of the Lamin B1 genomic binding profile affects rearrangement of heterochromatic domains and SAHF formation during senescence. Genes Dev 27, 1800–1808. 10.1101/gad.217281.113. 23964094 PMC3759696

[52] Shah PP, Donahue G, Otte GL, Capell BC, Nelson DM, Cao K, Aggarwala V, Cruickshanks HA, Rai TS, McBryan T, (2013). Lamin B1 depletion in senescent cells triggers large-scale changes in gene expression and the chromatin landscape. Genes Dev 27, 1787–1799. 10.1101/gad.223834.113. 23934658 PMC3759695

[53] Olan I, Parry AJ, Schoenfelder S, Narita M, Ito Y, Chan ASL, Slater GSC, Bihary D, Bando M, Shirahige K, (2020). Transcription-dependent cohesin repositioning rewires chromatin loops in cellular senescence. Nat Commun 11, 6049. 10.1038/s41467-020-19878-4. 33247104 PMC7695716

[54] Tasdemir N, Banito A, Roe JS, Alonso-Curbelo D, Camiolo M, Tschaharganeh DF, Huang CH, Aksoy O, Bolden JE, Chen CC, (2016). BRD4 Connects Enhancer Remodeling to Senescence Immune Surveillance. Cancer Discov 6, 612–629. 10.1158/2159-8290.Cd-16-0217. 27099234 PMC4893996

[55] Aird KM, Iwasaki O, Kossenkov AV, Tanizawa H, Fatkhutdinov N, Bitler BG, Le L, Alicea G, Yang TL, Johnson FB, (2016). HMGB2 orchestrates the chromatin landscape of senescence-associated secretory phenotype gene loci. J Cell Biol 215, 325–334. 10.1083/jcb.201608026. 27799366 PMC5100296

[56] Sen P, Lan Y, Li CY, Sidoli S, Donahue G, Dou Z, Frederick B, Chen Q, Luense LJ, Garcia BA, (2019). Histone Acetyltransferase p300 Induces De Novo Super-Enhancers to Drive Cellular Senescence. Mol Cell 73, 684–698.e688. 10.1016/j.molcel.2019.01.021. 30773298 PMC6688479

[57] Wang E. (1995). Senescent human fibroblasts resist programmed cell death, and failure to suppress bcl2 is involved. Cancer Res 55, 2284–2292. 7757977

[58] Di Micco R, Sulli G, Dobreva M, Liontos M, Botrugno OA, Gargiulo G, dal Zuffo R, Matti V, d'Ario G, Montani E, (2011). Interplay between oncogene-induced DNA damage response and heterochromatin in senescence and cancer. Nat Cell Biol 13, 292–302. 10.1038/ncb2170. 21336312 PMC3918344

[59] Chang J, Wang Y, Shao L, Laberge RM, Demaria M, Campisi J, Janakiraman K, Sharpless NE, Ding S, Feng W, (2016). Clearance of senescent cells by ABT263 rejuvenates aged hematopoietic stem cells in mice. Nat Med 22, 78–83. 10.1038/nm.4010. 26657143 PMC4762215

[60] Yosef R, Pilpel N, Tokarsky-Amiel R, Biran A, Ovadya Y, Cohen S, Vadai E, Dassa L, Shahar E, Condiotti R, (2016). Directed elimination of senescent cells by inhibition of BCL-W and BCL-XL. Nat Commun 7, 11190. 10.1038/ncomms11190. 27048913 PMC4823827

[61] Maus M, Lopez-Polo V, Mateo L, Lafarga M, Aguilera M, De Lama E, Meyer K, Sola A, Lopez-Martinez C, Lopez-Alonso I, (2023). Iron accumulation drives fibrosis, senescence and the senescence-associated secretory phenotype. Nat Metab 5, 2111–2130. 10.1038/s42255-023-00928-2. 38097808 PMC10730403

[62] Sun DY, Wu WB, Wu JJ, Shi Y, Xu JJ, Ouyang SX, Chi C, Shi Y, Ji QX, Miao JH, (2024). Pro-ferroptotic signaling promotes arterial aging via vascular smooth muscle cell senescence. Nat Commun 15, 1429. 10.1038/s41467-024-45823-w. 38365899 PMC10873425

[63] Feng Y, Wei H, Lyu M, Yu Z, Chen J, Lyu X, and Zhuang F. (2024). Iron retardation in lysosomes protects senescent cells from ferroptosis. Aging (Albany NY) 16, 7683–7703. 10.18632/aging.205777. 38683121 PMC11131988

[64] Wang B, Han J, Elisseeff JH, and Demaria M. (2024). The senescence-associated secretory phenotype and its physiological and pathological implications. Nat Rev Mol Cell Biol 25, 958–978. 10.1038/s41580-024-00727-x. 38654098

[65] Schafer MJ, White TA, Iijima K, Haak AJ, Ligresti G, Atkinson EJ, Oberg AL, Birch J, Salmonowicz H, Zhu Y, (2017). Cellular senescence mediates fibrotic pulmonary disease. Nat Commun 8, 14532. 10.1038/ncomms14532. 28230051 PMC5331226

[66] Bavik C, Coleman I, Dean JP, Knudsen B, Plymate S, and Nelson PS. (2006). The gene expression program of prostate fibroblast senescence modulates neoplastic epithelial cell proliferation through paracrine mechanisms. Cancer Res 66, 794–802. 10.1158/0008-5472.Can-05-1716. 16424011

[67] Hoare M, Ito Y, Kang TW, Weekes MP, Matheson NJ, Patten DA, Shetty S, Parry AJ, Menon S, Salama R, (2016). NOTCH1 mediates a switch between two distinct secretomes during senescence. Nat Cell Biol 18, 979–992. 10.1038/ncb3397. 27525720 PMC5008465

[68] Chen HA, Ho YJ, Mezzadra R, Adrover JM, Smolkin R, Zhu C, Woess K, Bernstein N, Schmitt G, Fong L, (2023). Senescence Rewires Microenvironment Sensing to Facilitate Antitumor Immunity. Cancer Discov 13, 432–453. 10.1158/2159-8290.Cd-22-0528. 36302222 PMC9901536

[69] Deng Y, Liu T, Scifo E, Li T, Xie K, Taschler B, Morsy S, Schaaf K, Ehninger A, Bano D, and Ehninger D. (2024). Analysis of the senescence-associated cell surfaceome reveals potential senotherapeutic targets. Aging Cell 23, e14312. 10.1111/acel.14312. 39228130 PMC11634743

[70] Amor C, Feucht J, Leibold J, Ho YJ, Zhu C, Alonso-Curbelo D, Mansilla-Soto J, Boyer JA, Li X, Giavridis T, (2020). Senolytic CAR T cells reverse senescence-associated pathologies. Nature 583, 127–132. 10.1038/s41586-020-2403-9. 32555459 PMC7583560

[71] Marin I, Boix O, Garcia-Garijo A, Sirois I, Caballe A, Zarzuela E, Ruano I, Attolini CS, Prats N, López-Domínguez JA, (2023). Cellular Senescence Is Immunogenic and Promotes Antitumor Immunity. Cancer Discov 13, 410–431. 10.1158/2159-8290.Cd-22-0523. 36302218 PMC7614152

[72] Teo YV, Rattanavirotkul N, Olova N, Salzano A, Quintanilla A, Tarrats N, Kiourtis C, Müller M, Green AR, Adams PD, (2019). Notch Signaling Mediates Secondary Senescence. Cell Rep 27, 997–1007.e1005. 10.1016/j.celrep.2019.03.104. 31018144 PMC6486482

[73] Parry AJ, Hoare M, Bihary D, Hänsel-Hertsch R, Smith S, Tomimatsu K, Mannion E, Smith A, D'Santos P, Russell IA, (2018). NOTCH-mediated non-cell autonomous regulation of chromatin structure during senescence. Nat Commun 9, 1840. 10.1038/s41467-018-04283-9. 29743479 PMC5943456

[74] Iltis C, Moskalevska I, Debiesse A, Seguin L, Fissoun C, Cervera L, Moudombi L, Ardin M, Ferrari A, Eliott C, (2025). A ganglioside-based immune checkpoint enables senescent cells to evade immunosurveillance during aging. Nat Aging 5, 219–236. 10.1038/s43587-024-00776-z. 39730825 PMC11839482

[75] Dou Z, Ghosh K, Vizioli MG, Zhu J, Sen P, Wangensteen KJ, Simithy J, Lan Y, Lin Y, Zhou Z, (2017). Cytoplasmic chromatin triggers inflammation in senescence and cancer. Nature 550, 402–406. 10.1038/nature24050. 28976970 PMC5850938

[76] Glück S, Guey B, Gulen MF, Wolter K, Kang TW, Schmacke NA, Bridgeman A, Rehwinkel J, Zender L, and Ablasser A. (2017). Innate immune sensing of cytosolic chromatin fragments through cGAS promotes senescence. Nat Cell Biol 19, 1061–1070. 10.1038/ncb3586. 28759028 PMC5826565

[77] Liu Y, Pagacz J, Wolfgeher DJ, Bromerg KD, Gorman JV, and Kron SJ. (2023). Senescent cancer cell vaccines induce cytotoxic T cell responses targeting primary tumors and disseminated tumor cells. J Immunother Cancer 11. 10.1136/jitc-2022-005862. PMC9933761 36792123

[78] Reimann M, Schrezenmeier J, Richter-Pechanska P, Dolnik A, Hick TP, Schleich K, Cai X, Fan DNY, Lohneis P, Maßwig S, (2021). Adaptive T-cell immunity controls senescence-prone MyD88- or CARD11-mutant B-cell lymphomas. Blood 137, 2785–2799. 10.1182/blood.2020005244. 33232972

[79] Belenki D, Richter-Pechanska P, Shao Z, Bhattacharya A, Lau A, Nabuco Leva Ferreira de Freitas JA, Kandler G, Hick TP, Cai X, Scharnagl E, (2025). Senescence-associated lineage-aberrant plasticity evokes T-cell-mediated tumor control. Nat Commun 16, 3079. 10.1038/s41467-025-57429-x. 40159497 PMC11955568

[80] Dörr JR, Yu Y, Milanovic M, Beuster G, Zasada C, Däbritz JH, Lisec J, Lenze D, Gerhardt A, Schleicher K, (2013). Synthetic lethal metabolic targeting of cellular senescence in cancer therapy. Nature 501, 421–425. 10.1038/nature12437. 23945590

[81] Quijano C, Cao L, Fergusson MM, Romero H, Liu J, Gutkind S, Rovira II, Mohney RP, Karoly ED, and Finkel T. (2012). Oncogene-induced senescence results in marked metabolic and bioenergetic alterations. Cell Cycle 11, 1383–1392. 10.4161/cc.19800. 22421146 PMC3350879

[82] Kaplon J, Zheng L, Meissl K, Chaneton B, Selivanov VA, Mackay G, van der Burg SH, Verdegaal EM, Cascante M, Shlomi T, (2013). A key role for mitochondrial gatekeeper pyruvate dehydrogenase in oncogene-induced senescence. Nature 498, 109–112. 10.1038/nature12154. 23685455

[83] Wiley CD, Velarde MC, Lecot P, Liu S, Sarnoski EA, Freund A, Shirakawa K, Lim HW, Davis SS, Ramanathan A, (2016). Mitochondrial Dysfunction Induces Senescence with a Distinct Secretory Phenotype. Cell Metab 23, 303–314. 10.1016/j.cmet.2015.11.011. 26686024 PMC4749409

[84] Zwerschke W, Mazurek S, Stöckl P, Hütter E, Eigenbrodt E, and Jansen-Dürr P. (2003). Metabolic analysis of senescent human fibroblasts reveals a role for AMP in cellular senescence. Biochem J 376, 403–411. 10.1042/bj20030816. 12943534 PMC1223775

[85] Wiley CD, Sharma R, Davis SS, Lopez-Dominguez JA, Mitchell KP, Wiley S, Alimirah F, Kim DE, Payne T, Rosko A, (2021). Oxylipin biosynthesis reinforces cellular senescence and allows detection of senolysis. Cell Metab 33, 1124–1136.e1125. 10.1016/j.cmet.2021.03.008. 33811820 PMC8501892

[86] Guerrero A, Herranz N, Sun B, Wagner V, Gallage S, Guiho R, Wolter K, Pombo J, Irvine EE, Innes AJ, (2019). Cardiac glycosides are broad-spectrum senolytics. Nat Metab 1, 1074–1088. 10.1038/s42255-019-0122-z. 31799499 PMC6887543

[87] Dimri GP, Lee X, Basile G, Acosta M, Scott G, Roskelley C, Medrano EE, Linskens M, Rubelj I, Pereira-Smith O, and (1995). A biomarker that identifies senescent human cells in culture and in aging skin in vivo. Proc Natl Acad Sci U S A 92, 9363–9367. 10.1073/pnas.92.20.9363. 7568133 PMC40985

[88] Lee BY, Han JA, Im JS, Morrone A, Johung K, Goodwin EC, Kleijer WJ, DiMaio D, and Hwang ES. (2006). Senescence-associated beta-galactosidase is lysosomal beta-galactosidase. Aging Cell 5, 187–195. 10.1111/j.1474-9726.2006.00199.x. 16626397

[89] Young AR, Narita M, Ferreira M, Kirschner K, Sadaie M, Darot JF, Tavaré S, Arakawa S, Shimizu S, Watt FM, and Narita M. (2009). Autophagy mediates the mitotic senescence transition. Genes Dev 23, 798–803. 10.1101/gad.519709. 19279323 PMC2666340

[90] Fuhrmann-Stroissnigg H, Ling YY, Zhao J, McGowan SJ, Zhu Y, Brooks RW, Grassi D, Gregg SQ, Stripay JL, Dorronsoro A, (2017). Identification of HSP90 inhibitors as a novel class of senolytics. Nat Commun 8, 422. 10.1038/s41467-017-00314-z. 28871086 PMC5583353

[91] McHugh D, Sun B, Gutierrez-Muñoz C, Hernández-González F, Mellone M, Guiho R, Duran I, Pombo J, Pietrocola F, Birch J, (2023). COPI vesicle formation and N-myristoylation are targetable vulnerabilities of senescent cells. Nat Cell Biol 25, 1804–1820. 10.1038/s41556-023-01287-6. 38012402 PMC10709147

[92] Sabath N, Levy-Adam F, Younis A, Rozales K, Meller A, Hadar S, Soueid-Baumgarten S, and Shalgi R. (2020). Cellular proteostasis decline in human senescence. Proc Natl Acad Sci U S A 117, 31902–31913. 10.1073/pnas.2018138117. 33257563 PMC7749315

[93] Johmura Y, Yamanaka T, Omori S, Wang TW, Sugiura Y, Matsumoto M, Suzuki N, Kumamoto S, Yamaguchi K, Hatakeyama S, (2021). Senolysis by glutaminolysis inhibition ameliorates various age-associated disorders. Science 371, 265–270. 10.1126/science.abb5916. 33446552

[94] Anerillas C, Mazan-Mamczarz K, Herman AB, Munk R, Lam KG, Calvo-Rubio M, Garrido A, Tsitsipatis D, Martindale JL, Altés G, (2023). The YAP-TEAD complex promotes senescent cell survival by lowering endoplasmic reticulum stress. Nat Aging 3, 1237–1250. 10.1038/s43587-023-00480-4. 37667102 PMC11369890

[95] Kurz DJ, Decary S, Hong Y, and Erusalimsky JD. (2000). Senescence-associated (beta)-galactosidase reflects an increase in lysosomal mass during replicative ageing of human endothelial cells. J Cell Sci 113 (Pt 20), 3613–3622. 10.1242/jcs.113.20.3613. 11017877

[96] Piechota M, Sunderland P, Wysocka A, Nalberczak M, Sliwinska MA, Radwanska K, and Sikora E. (2016). Is senescence-associated β-galactosidase a marker of neuronal senescence? Oncotarget 7, 81099–81109. 10.18632/oncotarget.12752. 27768595 PMC5348379

[97] Hsu CH, Altschuler SJ, and Wu LF. (2019). Patterns of Early p21 Dynamics Determine Proliferation-Senescence Cell Fate after Chemotherapy. Cell 178, 361–373.e312. 10.1016/j.cell.2019.05.041. 31204100 PMC6688629

[98] Demaria M, Ohtani N, Youssef SA, Rodier F, Toussaint W, Mitchell JR, Laberge RM, Vijg J, Van Steeg H, Dollé ME, (2014). An essential role for senescent cells in optimal wound healing through secretion of PDGF-AA. Dev Cell 31, 722–733. 10.1016/j.devcel.2014.11.012. 25499914 PMC4349629

[99] Lee S, Yu Y, Trimpert J, Benthani F, Mairhofer M, Richter-Pechanska P, Wyler E, Belenki D, Kaltenbrunner S, Pammer M, (2021). Virus-induced senescence is a driver and therapeutic target in COVID-19. Nature 599, 283–289. 10.1038/s41586-021-03995-1. 34517409

[100] Ashraf HM, Fernandez B, and Spencer SL. (2023). The intensities of canonical senescence biomarkers integrate the duration of cell-cycle withdrawal. Nat Commun 14, 4527. 10.1038/s41467-023-40132-0. 37500655 PMC10374620

[101] Reimann M, Lee S, and Schmitt CA. (2024). Cellular senescence: Neither irreversible nor reversible. J Exp Med 221. 10.1084/jem.20232136. PMC10883852 38385946

[102] Geradts J, Kratzke RA, Niehans GA, and Lincoln CE. (1995). Immunohistochemical detection of the cyclin-dependent kinase inhibitor 2/multiple tumor suppressor gene 1 (CDKN2/MTS1) product p16INK4A in archival human solid tumors: correlation with retinoblastoma protein expression. Cancer Res 55, 6006–6011. 8521382

[103] Gauthier ML, Berman HK, Miller C, Kozakeiwicz K, Chew K, Moore D, Rabban J, Chen YY, Kerlikowske K, and Tlsty TD. (2007). Abrogated response to cellular stress identifies DCIS associated with subsequent tumor events and defines basal-like breast tumors. Cancer Cell 12, 479–491. 10.1016/j.ccr.2007.10.017. 17996651 PMC3605202

[104] Simpson PT, Da Silva LM, and Lakhani SR. (2007). In situ carcinoma - can we predict which patient will come back with a recurrence? Cancer Cell 12, 409–411. 10.1016/j.ccr.2007.10.026. 17996643

[105] Kosar M, Bartkova J, Hubackova S, Hodny Z, Lukas J, and Bartek J. (2011). Senescence-associated heterochromatin foci are dispensable for cellular senescence, occur in a cell type- and insult-dependent manner and follow expression of p16(ink4a). Cell Cycle 10, 457–468. 10.4161/cc.10.3.14707. 21248468

[106] Sharpless NE, and Sherr CJ. (2015). Forging a signature of in vivo senescence. Nat Rev Cancer 15, 397–408. 10.1038/nrc3960. 26105537

[107] Saul D, Kosinsky RL, Atkinson EJ, Doolittle ML, Zhang X, LeBrasseur NK, Pignolo RJ, Robbins PD, Niedernhofer LJ, Ikeno Y, (2022). A new gene set identifies senescent cells and predicts senescence-associated pathways across tissues. Nat Commun 13, 4827. 10.1038/s41467-022-32552-1. 35974106 PMC9381717

[108] Jones RC, Karkanias J, Krasnow MA, Pisco AO, Quake SR, Salzman J, Yosef N, Bulthaup B, Brown P, Harper W, (2022). The Tabula Sapiens: A multiple-organ, single-cell transcriptomic atlas of humans. Science 376, eabl4896. 10.1126/science.abl4896. 35549404 PMC9812260

[109] Haston S, Gonzalez-Gualda E, Morsli S, Ge J, Reen V, Calderwood A, Moutsopoulos I, Panousopoulos L, Deletic P, Carreno G, (2023). Clearance of senescent macrophages ameliorates tumorigenesis in KRAS-driven lung cancer. Cancer Cell 41, 1242–1260.e1246. 10.1016/j.ccell.2023.05.004. 37267953

[110] Prieto LI, Sturmlechner I, Graves SI, Zhang C, Goplen NP, Yi ES, Sun J, Li H, and Baker DJ. (2023). Senescent alveolar macrophages promote early-stage lung tumorigenesis. Cancer Cell 41, 1261–1275.e1266. 10.1016/j.ccell.2023.05.006. 37267954 PMC10524974

[111] Behmoaras J, and Gil J. (2021). Similarities and interplay between senescent cells and macrophages. J Cell Biol 220. 10.1083/jcb.202010162. PMC7769159 33355620

[112] Gurkar AU, Gerencser AA, Mora AL, Nelson AC, Zhang AR, Lagnado AB, Enninful A, Benz C, Furman D, Beaulieu D, (2023). Spatial mapping of cellular senescence: emerging challenges and opportunities. Nat Aging 3, 776–790. 10.1038/s43587-023-00446-6. 37400722 PMC10505496

[113] Ma S, Ji Z, Zhang B, Geng L, Cai Y, Nie C, Li J, Zuo Y, Sun Y, Xu G, (2024). Spatial transcriptomic landscape unveils immunoglobin-associated senescence as a hallmark of aging. Cell 187, 7025–7044.e7034. 10.1016/j.cell.2024.10.019. 39500323

[114] Hanahan D, and Weinberg RA. (2011). Hallmarks of cancer: the next generation. Cell 144, 646–674. 10.1016/j.cell.2011.02.013. 21376230

[115] Hanahan D. (2022). Hallmarks of Cancer: New Dimensions. Cancer Discov 12, 31–46. 10.1158/2159-8290.Cd-21-1059. 35022204

[116] Hanahan D. (2026). Hallmarks of cancer-Then and now, and beyond. Cell. 10.1016/j.cell.2025.12.049.

[117] Herranz N, and Gil J. (2018). Mechanisms and functions of cellular senescence. J Clin Invest 128, 1238–1246. 10.1172/jci95148. 29608137 PMC5873888

[118] d'Adda di Fagagna F, Reaper PM, Clay-Farrace L, Fiegler H, Carr P, Von Zglinicki T, Saretzki G, Carter NP, and Jackson SP. (2003). A DNA damage checkpoint response in telomere-initiated senescence. Nature 426, 194–198. 10.1038/nature02118. 14608368

[119] Fumagalli M, Rossiello F, Clerici M, Barozzi S, Cittaro D, Kaplunov JM, Bucci G, Dobreva M, Matti V, Beausejour CM, (2012). Telomeric DNA damage is irreparable and causes persistent DNA-damage-response activation. Nat Cell Biol 14, 355–365. 10.1038/ncb2466. 22426077 PMC3717580

[120] te Poele RH, Okorokov AL, Jardine L, Cummings J, and Joel SP. (2002). DNA damage is able to induce senescence in tumor cells in vitro and in vivo. Cancer Res 62, 1876–1883. 11912168

[121] Di Micco R, Fumagalli M, Cicalese A, Piccinin S, Gasparini P, Luise C, Schurra C, Garre M, Nuciforo PG, Bensimon A, (2006). Oncogene-induced senescence is a DNA damage response triggered by DNA hyper-replication. Nature 444, 638–642. 10.1038/nature05327. 17136094

[122] Kamijo T, Zindy F, Roussel MF, Quelle DE, Downing JR, Ashmun RA, Grosveld G, and Sherr CJ. (1997). Tumor suppression at the mouse INK4a locus mediated by the alternative reading frame product p19ARF. Cell 91, 649–659. 10.1016/s0092-8674(00)80452-3. 9393858

[123] de Stanchina E, McCurrach ME, Zindy F, Shieh SY, Ferbeyre G, Samuelson AV, Prives C, Roussel MF, Sherr CJ, and Lowe SW. (1998). E1A signaling to p53 involves the p19(ARF) tumor suppressor. Genes Dev 12, 2434–2442. 10.1101/gad.12.15.2434. 9694807 PMC317046

[124] Sherr CJ. (2001). The INK4a/ARF network in tumour suppression. Nat Rev Mol Cell Biol 2, 731–737. 10.1038/35096061. 11584300

[125] Bates S, and Vousden KH. (1999). Mechanisms of p53-mediated apoptosis. Cell Mol Life Sci 55, 28–37. 10.1007/s000180050267. 10065149 PMC11147148

[126] Sherr CJ, and McCormick F. (2002). The RB and p53 pathways in cancer. Cancer Cell 2, 103–112. 10.1016/s1535-6108(02)00102-2. 12204530

[127] Oruetxebarria I, Venturini F, Kekarainen T, Houweling A, Zuijderduijn LM, Mohd-Sarip A, Vries RG, Hoeben RC, and Verrijzer CP. (2004). P16INK4a is required for hSNF5 chromatin remodeler-induced cellular senescence in malignant rhabdoid tumor cells. J Biol Chem 279, 3807–3816. 10.1074/jbc.M309333200. 14604992

[128] Gil J, and Peters G. (2006). Regulation of the INK4b-ARF-INK4a tumour suppressor locus: all for one or one for all. Nat Rev Mol Cell Biol 7, 667–677. 10.1038/nrm1987. 16921403

[129] Bracken AP, Kleine-Kohlbrecher D, Dietrich N, Pasini D, Gargiulo G, Beekman C, Theilgaard-Mönch K, Minucci S, Porse BT, Marine JC, (2007). The Polycomb group proteins bind throughout the INK4A-ARF locus and are disassociated in senescent cells. Genes Dev 21, 525–530. 10.1101/gad.415507. 17344414 PMC1820894

[130] Gil J, Bernard D, Martínez D, and Beach D. (2004). Polycomb CBX7 has a unifying role in cellular lifespan. Nat Cell Biol 6, 67–72. 10.1038/ncb1077. 14647293

[131] Agger K, Cloos PA, Rudkjaer L, Williams K, Andersen G, Christensen J, and Helin K. (2009). The H3K27me3 demethylase JMJD3 contributes to the activation of the INK4A-ARF locus in response to oncogene- and stress-induced senescence. Genes Dev 23, 1171–1176. 10.1101/gad.510809. 19451217 PMC2685535

[132] Jacobs JJ, Kieboom K, Marino S, DePinho RA, and van Lohuizen M. (1999). The oncogene and Polycomb-group gene bmi-1 regulates cell proliferation and senescence through the ink4a locus. Nature 397, 164–168. 10.1038/16476. 9923679

[133] Yap KL, Li S, Muñoz-Cabello AM, Raguz S, Zeng L, Mujtaba S, Gil J, Walsh MJ, and Zhou MM. (2010). Molecular interplay of the noncoding RNA ANRIL and methylated histone H3 lysine 27 by polycomb CBX7 in transcriptional silencing of INK4a. Mol Cell 38, 662–674. 10.1016/j.molcel.2010.03.021. 20541999 PMC2886305

[134] Good L, Dimri GP, Campisi J, and Chen KY. (1996). Regulation of dihydrofolate reductase gene expression and E2F components in human diploid fibroblasts during growth and senescence. J Cell Physiol 168, 580–588. 10.1002/(sici)1097-4652(199609)168:3<580::Aid-jcp10>3.0.Co;2-3. 8816912

[135] Chen T, Xue L, Niu J, Ma L, Li N, Cao X, Li Q, Wang M, Zhao W, Li G, (2012). The retinoblastoma protein selectively represses E2F1 targets via a TAAC DNA element during cellular senescence. J Biol Chem 287, 37540–37551. 10.1074/jbc.M111.260679. 22955272 PMC3481348

[136] Vernier M, Bourdeau V, Gaumont-Leclerc MF, Moiseeva O, Bégin V, Saad F, Mes-Masson AM, and Ferbeyre G. (2011). Regulation of E2Fs and senescence by PML nuclear bodies. Genes Dev 25, 41–50. 10.1101/gad.1975111. 21205865 PMC3012935

[137] Tomimatsu K, Bihary D, Olan I, Parry AJ, Schoenfelder S, Chan ASL, Slater GSC, Ito Y, Rugg-Gunn PJ, Kirschner K, (2022). Locus-specific induction of gene expression from heterochromatin loci during cellular senescence. Nat Aging 2, 31–45. 10.1038/s43587-021-00147-y. 37118356

[138] Olan I, Ando-Kuri M, Parry AJ, Handa T, Schoenfelder S, Fraser P, Ohkawa Y, Kimura H, Narita M, and Narita M. (2024). HMGA1 orchestrates chromatin compartmentalization and sequesters genes into 3D networks coordinating senescence heterogeneity. Nat Commun 15, 6891. 10.1038/s41467-024-51153-8. 39134516 PMC11319441

[139] Sidler C, Woycicki R, Li D, Wang B, Kovalchuk I, and Kovalchuk O. (2014). A role for SUV39H1-mediated H3K9 trimethylation in the control of genome stability and senescence in WI38 human diploid lung fibroblasts. Aging (Albany NY) 6, 545–563. 10.18632/aging.100678. 25063769 PMC4153622

[140] Lopes-Paciencia S, Bourdeau V, Rowell MC, Amirimehr D, Guillon J, Kalegari P, Barua A, Quoc-Huy Trinh V, Azzi F, Turcotte S, (2024). A senescence restriction point acting on chromatin integrates oncogenic signals. Cell Rep 43, 114044. 10.1016/j.celrep.2024.114044. 38568812

[141] Chien Y, Scuoppo C, Wang X, Fang X, Balgley B, Bolden JE, Premsrirut P, Luo W, Chicas A, Lee CS, (2011). Control of the senescence-associated secretory phenotype by NF-κB promotes senescence and enhances chemosensitivity. Genes Dev 25, 2125–2136. 10.1101/gad.17276711. 21979375 PMC3205583

[142] Kang C, Xu Q, Martin TD, Li MZ, Demaria M, Aron L, Lu T, Yankner BA, Campisi J, and Elledge SJ. (2015). The DNA damage response induces inflammation and senescence by inhibiting autophagy of GATA4. Science 349, aaa5612. 10.1126/science.aaa5612. 26404840 PMC4942138

[143] Birch J, and Gil J. (2020). Senescence and the SASP: many therapeutic avenues. Genes Dev 34, 1565–1576. 10.1101/gad.343129.120. 33262144 PMC7706700

[144] Faget DV, Ren Q, and Stewart SA. (2019). Unmasking senescence: context-dependent effects of SASP in cancer. Nat Rev Cancer 19, 439–453. 10.1038/s41568-019-0156-2. 31235879

[145] Zhang L, Yang P, Chen J, Chen Z, Liu Z, Feng G, Sha F, Li Z, Xu Z, Huang Y, (2023). CD44 connects autophagy decline and ageing in the vascular endothelium. Nat Commun 14, 5524. 10.1038/s41467-023-41346-y. 37684253 PMC10491636

[146] Gorgoulis VG, Zacharatos P, Kotsinas A, Kletsas D, Mariatos G, Zoumpourlis V, Ryan KM, Kittas C, and Papavassiliou AG. (2003). p53 activates ICAM-1 (CD54) expression in an NF-kappaB-independent manner. Embo j 22, 1567–1578. 10.1093/emboj/cdg157. 12660163 PMC152901

[147] Guillon J, Petit C, Moreau M, Toutain B, Henry C, Roché H, Bonichon-Lamichhane N, Salmon JP, Lemonnier J, Campone M, (2019). Regulation of senescence escape by TSP1 and CD47 following chemotherapy treatment. Cell Death Dis 10, 199. 10.1038/s41419-019-1406-7. 30814491 PMC6393582

[148] Muñoz DP, Yannone SM, Daemen A, Sun Y, Vakar-Lopez F, Kawahara M, Freund AM, Rodier F, Wu JD, Desprez PY, (2019). Targetable mechanisms driving immunoevasion of persistent senescent cells link chemotherapy-resistant cancer to aging. JCI Insight 5. 10.1172/jci.insight.124716. PMC6675550 31184599

[149] Onorati A, Havas AP, Lin B, Rajagopal J, Sen P, Adams PD, and Dou Z. (2022). Upregulation of PD-L1 in Senescence and Aging. Mol Cell Biol 42, e0017122. 10.1128/mcb.00171-22. 36154662 PMC9583718

[150] Ito T, Teo YV, Evans SA, Neretti N, and Sedivy JM. (2018). Regulation of Cellular Senescence by Polycomb Chromatin Modifiers through Distinct DNA Damage- and Histone Methylation-Dependent Pathways. Cell Rep 22, 3480–3492. 10.1016/j.celrep.2018.03.002. 29590617 PMC5915310

[151] Herranz N, Gallage S, Mellone M, Wuestefeld T, Klotz S, Hanley CJ, Raguz S, Acosta JC, Innes AJ, Banito A, (2015). mTOR regulates MAPKAPK2 translation to control the senescence-associated secretory phenotype. Nat Cell Biol 17, 1205–1217. 10.1038/ncb3225. 26280535 PMC4589897

[152] Laberge RM, Sun Y, Orjalo AV, Patil CK, Freund A, Zhou L, Curran SC, Davalos AR, Wilson-Edell KA, Liu S, (2015). MTOR regulates the pro-tumorigenic senescence-associated secretory phenotype by promoting IL1A translation. Nat Cell Biol 17, 1049–1061. 10.1038/ncb3195. 26147250 PMC4691706

[153] Freund A, Patil CK, and Campisi J. (2011). p38MAPK is a novel DNA damage response-independent regulator of the senescence-associated secretory phenotype. EMBO J 30, 1536–1548. 10.1038/emboj.2011.69. 21399611 PMC3102277

[154] De Cecco M, Ito T, Petrashen AP, Elias AE, Skvir NJ, Criscione SW, Caligiana A, Brocculi G, Adney EM, Boeke JD, (2019). L1 drives IFN in senescent cells and promotes age-associated inflammation. Nature 566, 73–78. 10.1038/s41586-018-0784-9. 30728521 PMC6519963

[155] Wiley CD, and Campisi J. (2021). The metabolic roots of senescence: mechanisms and opportunities for intervention. Nat Metab 3, 1290–1301. 10.1038/s42255-021-00483-8. 34663974 PMC8889622

[156] Sun X, Li Q, Tang Y, Hu W, Chen G, An H, Huang D, Tong T, and Zhang Y. (2023). Epigenetic activation of secretory phenotypes in senescence by the FOXQ1-SIRT4-GDH signaling. Cell Death Dis 14, 481. 10.1038/s41419-023-06002-9. 37516739 PMC10387070

[157] Vogelstein B, Papadopoulos N, Velculescu VE, Zhou S, Diaz LA Jr., and Kinzler KW. (2013). Cancer genome landscapes. Science 339, 1546–1558. 10.1126/science.1235122. 23539594 PMC3749880

[158] Bell RJ, Rube HT, Xavier-Magalhães A, Costa BM, Mancini A, Song JS, and Costello JF. (2016). Understanding TERT Promoter Mutations: A Common Path to Immortality. Mol Cancer Res 14, 315–323. 10.1158/1541-7786.Mcr-16-0003. 26941407 PMC4852159

[159] Engeland K. (2022). Cell cycle regulation: p53-p21-RB signaling. Cell Death Differ 29, 946–960. 10.1038/s41418-022-00988-z. 35361964 PMC9090780

[160] Lin AW, Barradas M, Stone JC, van Aelst L, Serrano M, and Lowe SW. (1998). Premature senescence involving p53 and p16 is activated in response to constitutive MEK/MAPK mitogenic signaling. Genes Dev 12, 3008–3019. 10.1101/gad.12.19.3008. 9765203 PMC317198

[161] Wang W, Chen JX, Liao R, Deng Q, Zhou JJ, Huang S, and Sun P. (2002). Sequential activation of the MEK-extracellular signal-regulated kinase and MKK3/6-p38 mitogen-activated protein kinase pathways mediates oncogenic ras-induced premature senescence. Mol Cell Biol 22, 3389–3403. 10.1128/mcb.22.10.3389-3403.2002. 11971971 PMC133789

[162] Iwasa H, Han J, and Ishikawa F. (2003). Mitogen-activated protein kinase p38 defines the common senescence-signalling pathway. Genes Cells 8, 131–144. 10.1046/j.1365-2443.2003.00620.x. 12581156

[163] Bulavin DV, Kovalsky O, Hollander MC, and Fornace AJ Jr. (2003). Loss of oncogenic H-ras-induced cell cycle arrest and p38 mitogen-activated protein kinase activation by disruption of Gadd45a. Mol Cell Biol 23, 3859–3871. 10.1128/mcb.23.11.3859-3871.2003. 12748288 PMC155214

[164] Michaloglou C, Vredeveld LC, Soengas MS, Denoyelle C, Kuilman T, van der Horst CM, Majoor DM, Shay JW, Mooi WJ, and Peeper DS. (2005). BRAFE600-associated senescence-like cell cycle arrest of human naevi. Nature 436, 720–724. 10.1038/nature03890. 16079850

[165] Bartkova J, Rezaei N, Liontos M, Karakaidos P, Kletsas D, Issaeva N, Vassiliou LV, Kolettas E, Niforou K, Zoumpourlis VC, (2006). Oncogene-induced senescence is part of the tumorigenesis barrier imposed by DNA damage checkpoints. Nature 444, 633–637. 10.1038/nature05268. 17136093

[166] Zhu J, Woods D, McMahon M, and Bishop JM. (1998). Senescence of human fibroblasts induced by oncogenic Raf. Genes Dev 12, 2997–3007. 10.1101/gad.12.19.2997. 9765202 PMC317194

[167] Garcia-Cao I, Song MS, Hobbs RM, Laurent G, Giorgi C, de Boer VC, Anastasiou D, Ito K, Sasaki AT, Rameh L, (2012). Systemic elevation of PTEN induces a tumor-suppressive metabolic state. Cell 149, 49–62. 10.1016/j.cell.2012.02.030. 22401813 PMC3319228

[168] Braig M, Lee S, Loddenkemper C, Rudolph C, Peters AH, Schlegelberger B, Stein H, Dörken B, Jenuwein T, and Schmitt CA. (2005). Oncogene-induced senescence as an initial barrier in lymphoma development. Nature 436, 660–665. 10.1038/nature03841. 16079837

[169] Guerra C, Mijimolle N, Dhawahir A, Dubus P, Barradas M, Serrano M, Campuzano V, and Barbacid M. (2003). Tumor induction by an endogenous K-ras oncogene is highly dependent on cellular context. Cancer Cell 4, 111–120. 10.1016/s1535-6108(03)00191-0. 12957286

[170] Collado M, Gil J, Efeyan A, Guerra C, Schuhmacher AJ, Barradas M, Benguria A, Zaballos A, Flores JM, Barbacid M, (2005). Tumour biology: senescence in premalignant tumours. Nature 436, 642. 10.1038/436642a. 16079833

[171] Chen Z, Trotman LC, Shaffer D, Lin HK, Dotan ZA, Niki M, Koutcher JA, Scher HI, Ludwig T, Gerald W, (2005). Crucial role of p53-dependent cellular senescence in suppression of Pten-deficient tumorigenesis. Nature 436, 725–730. 10.1038/nature03918. 16079851 PMC1939938

[172] Sanchez-Vega F, Mina M, Armenia J, Chatila WK, Luna A, La KC, Dimitriadoy S, Liu DL, Kantheti HS, Saghafinia S, (2018). Oncogenic Signaling Pathways in The Cancer Genome Atlas. Cell 173, 321–337.e310. 10.1016/j.cell.2018.03.035. 29625050 PMC6070353

[173] Tuveson DA, Shaw AT, Willis NA, Silver DP, Jackson EL, Chang S, Mercer KL, Grochow R, Hock H, Crowley D, (2004). Endogenous oncogenic K-ras(G12D) stimulates proliferation and widespread neoplastic and developmental defects. Cancer Cell 5, 375–387. 10.1016/s1535-6108(04)00085-6. 15093544

[174] Junttila MR, Karnezis AN, Garcia D, Madriles F, Kortlever RM, Rostker F, Brown Swigart L, Pham DM, Seo Y, Evan GI, and Martins CP. (2010). Selective activation of p53-mediated tumour suppression in high-grade tumours. Nature 468, 567–571. 10.1038/nature09526. 21107427 PMC3011233

[175] Feldser DM, Kostova KK, Winslow MM, Taylor SE, Cashman C, Whittaker CA, Sanchez-Rivera FJ, Resnick R, Bronson R, Hemann MT, and Jacks T. (2010). Stage-specific sensitivity to p53 restoration during lung cancer progression. Nature 468, 572–575. 10.1038/nature09535. 21107428 PMC3003305

[176] Chan ASL, Zhu H, Narita M, Cassidy LD, Young ARJ, Bermejo-Rodriguez C, Janowska AT, Chen HC, Gough S, Oshimori N, (2024). Titration of RAS alters senescent state and influences tumour initiation. Nature 633, 678–685. 10.1038/s41586-024-07797-z. 39112713 PMC11410659

[177] Schlesinger Y, Yosefov-Levi O, Kolodkin-Gal D, Granit RZ, Peters L, Kalifa R, Xia L, Nasereddin A, Shiff I, Amran O, (2020). Single-cell transcriptomes of pancreatic preinvasive lesions and cancer reveal acinar metaplastic cells' heterogeneity. Nat Commun 11, 4516. 10.1038/s41467-020-18207-z. 32908137 PMC7481797

[178] Hoi XP, Stangis MM, Glass SE, Kim JH, Kang SW, Brennen WN, Li Z, Grady WM, Yegnasubramanian S, Kuhn P, (2025). Cellular senescence in precancer lesions and early-stage cancers. Cancer Cell. 10.1016/j.ccell.2025.10.006. PMC13425571 41202812

[179] Banito A, Rashid ST, Acosta JC, Li S, Pereira CF, Geti I, Pinho S, Silva JC, Azuara V, Walsh M, (2009). Senescence impairs successful reprogramming to pluripotent stem cells. Genes Dev 23, 2134–2139. 10.1101/gad.1811609. 19696146 PMC2751980

[180] Mosteiro L, Pantoja C, Alcazar N, Marión RM, Chondronasiou D, Rovira M, Fernandez-Marcos PJ, Muñoz-Martin M, Blanco-Aparicio C, Pastor J, (2016). Tissue damage and senescence provide critical signals for cellular reprogramming in vivo. Science 354. 10.1126/science.aaf4445. 27884981

[181] Soriani A, Zingoni A, Cerboni C, Iannitto ML, Ricciardi MR, Di Gialleonardo V, Cippitelli M, Fionda C, Petrucci MT, Guarini A, (2009). ATM-ATR-dependent up-regulation of DNAM-1 and NKG2D ligands on multiple myeloma cells by therapeutic agents results in enhanced NK-cell susceptibility and is associated with a senescent phenotype. Blood 113, 3503–3511. 10.1182/blood-2008-08-173914. 19098271

[182] Sagiv A, Burton DG, Moshayev Z, Vadai E, Wensveen F, Ben-Dor S, Golani O, Polic B, and Krizhanovsky V. (2016). NKG2D ligands mediate immunosurveillance of senescent cells. Aging (Albany NY) 8, 328–344. 10.18632/aging.100897. 26878797 PMC4789586

[183] Tahara H, Kamada K, Sato E, Tsuyama N, Kim JK, Hara E, Oda K, and Ide T. (1995). Increase in expression levels of interferon-inducible genes in senescent human diploid fibroblasts and in SV40-transformed human fibroblasts with extended lifespan. Oncogene 11, 1125–1132. 7566972

[184] Moiseeva O, Mallette FA, Mukhopadhyay UK, Moores A, and Ferbeyre G. (2006). DNA damage signaling and p53-dependent senescence after prolonged beta-interferon stimulation. Mol Biol Cell 17, 1583–1592. 10.1091/mbc.e05-09-0858. 16436515 PMC1415317

[185] Barriga FM, Tsanov KM, Ho YJ, Sohail N, Zhang A, Baslan T, Wuest AN, Del Priore I, Meškauskaitė B, Livshits G, (2022). MACHETE identifies interferon-encompassing chromosome 9p21.3 deletions as mediators of immune evasion and metastasis. Nat Cancer 3, 1367–1385. 10.1038/s43018-022-00443-5. 36344707 PMC9701143

[186] Ruscetti M, Leibold J, Bott MJ, Fennell M, Kulick A, Salgado NR, Chen CC, Ho YJ, Sanchez-Rivera FJ, Feucht J, (2018). NK cell-mediated cytotoxicity contributes to tumor control by a cytostatic drug combination. Science 362, 1416–1422. 10.1126/science.aas9090. 30573629 PMC6711172

[187] Lujambio A, Akkari L, Simon J, Grace D, Tschaharganeh DF, Bolden JE, Zhao Z, Thapar V, Joyce JA, Krizhanovsky V, and Lowe SW. (2013). Non-cell-autonomous tumor suppression by p53. Cell 153, 449–460. 10.1016/j.cell.2013.03.020. 23562644 PMC3702034

[188] Sturmlechner I, Zhang C, Sine CC, van Deursen EJ, Jeganathan KB, Hamada N, Grasic J, Friedman D, Stutchman JT, Can I, (2021). p21 produces a bioactive secretome that places stressed cells under immunosurveillance. Science 374, eabb3420. 10.1126/science.abb3420. 34709885 PMC8985214

[189] Debiesse A, Moudombi L, Voissière A, Lefèvre L, Malfroy M, Moskalevska I, Pereira Abrantes M, Vanacker H, Hubert M, Pommier R, (2025). Oncogenic Stress is a Novel Immunogenic Signal Driven by the Unfolded Protein Response and Detected by Neutrophils. bioRxiv, 2025.2001.2017.633444. 10.1101/2025.01.17.633444.

[190] Eggert T, Wolter K, Ji J, Ma C, Yevsa T, Klotz S, Medina-Echeverz J, Longerich T, Forgues M, Reisinger F, (2016). Distinct Functions of Senescence-Associated Immune Responses in Liver Tumor Surveillance and Tumor Progression. Cancer Cell 30, 533–547. 10.1016/j.ccell.2016.09.003. 27728804 PMC7789819

[191] Marin I, Serrano M, and Pietrocola F. (2023). Recent insights into the crosstalk between senescent cells and CD8 T lymphocytes. NPJ Aging 9, 8. 10.1038/s41514-023-00105-5. 37015935 PMC10073090

[192] Meng Y, Efimova EV, Hamzeh KW, Darga TE, Mauceri HJ, Fu YX, Kron SJ, and Weichselbaum RR. (2012). Radiation-inducible immunotherapy for cancer: senescent tumor cells as a cancer vaccine. Mol Ther 20, 1046–1055. 10.1038/mt.2012.19. 22334019 PMC3345982

[193] Cruickshanks HA, McBryan T, Nelson DM, Vanderkraats ND, Shah PP, van Tuyn J, Singh Rai T, Brock C, Donahue G, Dunican DS, (2013). Senescent cells harbour features of the cancer epigenome. Nat Cell Biol 15, 1495–1506. 10.1038/ncb2879. 24270890 PMC4106249

[194] Kinker GS, Greenwald AC, Tal R, Orlova Z, Cuoco MS, McFarland JM, Warren A, Rodman C, Roth JA, Bender SA, (2020). Pan-cancer single-cell RNA-seq identifies recurring programs of cellular heterogeneity. Nat Genet 52, 1208–1218. 10.1038/s41588-020-00726-6. 33128048 PMC8135089

[195] Johnstone SE, Reyes A, Qi Y, Adriaens C, Hegazi E, Pelka K, Chen JH, Zou LS, Drier Y, Hecht V, (2020). Large-Scale Topological Changes Restrain Malignant Progression in Colorectal Cancer. Cell 182, 1474–1489.e1423. 10.1016/j.cell.2020.07.030. 32841603 PMC7575124

[196] Alonso-Curbelo D, Ho YJ, Burdziak C, Maag JLV, Morris J.P.t., Chandwani R, Chen HA, Tsanov KM, Barriga FM, Luan W, (2021). A gene-environment-induced epigenetic program initiates tumorigenesis. Nature 590, 642–648. 10.1038/s41586-020-03147-x. 33536616 PMC8482641

[197] Jochems F, Thijssen B, De Conti G, Jansen R, Pogacar Z, Groot K, Wang L, Schepers A, Wang C, Jin H, (2021). The Cancer SENESCopedia: A delineation of cancer cell senescence. Cell Rep 36, 109441. 10.1016/j.celrep.2021.109441. 34320349 PMC8333195

[198] Malkin D. (2011). Li-fraumeni syndrome. Genes Cancer 2, 475–484. 10.1177/1947601911413466. 21779515 PMC3135649

[199] Soura E, Eliades PJ, Shannon K, Stratigos AJ, and Tsao H. (2016). Hereditary melanoma: Update on syndromes and management: Genetics of familial atypical multiple mole melanoma syndrome. J Am Acad Dermatol 74, 395–407; quiz 408–310. 10.1016/j.jaad.2015.08.038. 26892650 PMC4761105

[200] Draper GJ, Sanders BM, and Kingston JE. (1986). Second primary neoplasms in patients with retinoblastoma. Br J Cancer 53, 661–671. 10.1038/bjc.1986.110. 3718823 PMC2001387

[201] Storer M, Mas A, Robert-Moreno A, Pecoraro M, Ortells MC, Di Giacomo V, Yosef R, Pilpel N, Krizhanovsky V, Sharpe J, and Keyes WM. (2013). Senescence is a developmental mechanism that contributes to embryonic growth and patterning. Cell 155, 1119–1130. 10.1016/j.cell.2013.10.041. 24238961

[202] Muñoz-Espín D, Cañamero M, Maraver A, Gómez-López G, Contreras J, Murillo-Cuesta S, Rodríguez-Baeza A, Varela-Nieto I, Ruberte J, Collado M, and Serrano M. (2013). Programmed cell senescence during mammalian embryonic development. Cell 155, 1104–1118. 10.1016/j.cell.2013.10.019. 24238962

[203] Zhao Y, Tyshkovskiy A, Muñoz-Espín D, Tian X, Serrano M, de Magalhaes JP, Nevo E, Gladyshev VN, Seluanov A, and Gorbunova V. (2018). Naked mole rats can undergo developmental, oncogene-induced and DNA damage-induced cellular senescence. Proc Natl Acad Sci U S A 115, 1801–1806. 10.1073/pnas.1721160115. 29432174 PMC5828632

[204] Da Silva-Álvarez S, Guerra-Varela J, Sobrido-Cameán D, Quelle A, Barreiro-Iglesias A, Sánchez L, and Collado M. (2020). Developmentally-programmed cellular senescence is conserved and widespread in zebrafish. Aging (Albany NY) 12, 17895–17901. 10.18632/aging.103968. 32991320 PMC7585104

[205] Li Y, Zhao H, Huang X, Tang J, Zhang S, Li Y, Liu X, He L, Ju Z, Lui KO, and Zhou B. (2018). Embryonic senescent cells re-enter cell cycle and contribute to tissues after birth. Cell Res 28, 775–778. 10.1038/s41422-018-0050-6. 29872106 PMC6028486

[206] Reyes NS, Krasilnikov M, Allen NC, Lee JY, Hyams B, Zhou M, Ravishankar S, Cassandras M, Wang C, Khan I, (2022). Sentinel p16(INK4a+) cells in the basement membrane form a reparative niche in the lung. Science 378, 192–201. 10.1126/science.abf3326. 36227993 PMC10621323

[207] Grosse L, Wagner N, Emelyanov A, Molina C, Lacas-Gervais S, Wagner KD, and Bulavin DV. (2020). Defined p16(High) Senescent Cell Types Are Indispensable for Mouse Healthspan. Cell Metab 32, 87–99.e86. 10.1016/j.cmet.2020.05.002. 32485135

[208] Zhao H, Liu Z, Chen H, Han M, Zhang M, Liu K, Jin H, Liu X, Shi M, Pu W, (2024). Identifying specific functional roles for senescence across cell types. Cell 187, 7314–7334.e7321. 10.1016/j.cell.2024.09.021. 39368477

[209] Acosta JC, Banito A, Wuestefeld T, Georgilis A, Janich P, Morton JP, Athineos D, Kang TW, Lasitschka F, Andrulis M, (2013). A complex secretory program orchestrated by the inflammasome controls paracrine senescence. Nat Cell Biol 15, 978–990. 10.1038/ncb2784. 23770676 PMC3732483

[210] Chiche A, Le Roux I, von Joest M, Sakai H, Aguín SB, Cazin C, Salam R, Fiette L, Alegria O, Flamant P, (2017). Injury-Induced Senescence Enables In Vivo Reprogramming in Skeletal Muscle. Cell Stem Cell 20, 407–414.e404. 10.1016/j.stem.2016.11.020. 28017795

[211] MacCarthy-Morrogh L, and Martin P. (2020). The hallmarks of cancer are also the hallmarks of wound healing. Sci Signal 13. 10.1126/scisignal.aay8690. 32900881

[212] Ansieau S, Bastid J, Doreau A, Morel AP, Bouchet BP, Thomas C, Fauvet F, Puisieux I, Doglioni C, Piccinin S, (2008). Induction of EMT by twist proteins as a collateral effect of tumor-promoting inactivation of premature senescence. Cancer Cell 14, 79–89. 10.1016/j.ccr.2008.06.005. 18598946

[213] Milanovic M, Fan DNY, Belenki D, Däbritz JHM, Zhao Z, Yu Y, Dörr JR, Dimitrova L, Lenze D, Monteiro Barbosa IA, (2018). Senescence-associated reprogramming promotes cancer stemness. Nature 553, 96–100. 10.1038/nature25167. 29258294

[214] Zhang L, Pitcher LE, Yousefzadeh MJ, Niedernhofer LJ, Robbins PD, and Zhu Y. (2022). Cellular senescence: a key therapeutic target in aging and diseases. J Clin Invest 132. 10.1172/jci158450. PMC9337830 35912854

[215] Jeon OH, Kim C, Laberge RM, Demaria M, Rathod S, Vasserot AP, Chung JW, Kim DH, Poon Y, David N, (2017). Local clearance of senescent cells attenuates the development of post-traumatic osteoarthritis and creates a pro-regenerative environment. Nat Med 23, 775–781. 10.1038/nm.4324. 28436958 PMC5785239

[216] Ogrodnik M, Miwa S, Tchkonia T, Tiniakos D, Wilson CL, Lahat A, Day CP, Burt A, Palmer A, Anstee QM, (2017). Cellular senescence drives age-dependent hepatic steatosis. Nat Commun 8, 15691. 10.1038/ncomms15691. 28608850 PMC5474745

[217] Bussian TJ, Aziz A, Meyer CF, Swenson BL, van Deursen JM, and Baker DJ. (2018). Clearance of senescent glial cells prevents tau-dependent pathology and cognitive decline. Nature 562, 578–582. 10.1038/s41586-018-0543-y. 30232451 PMC6206507

[218] Childs BG, Baker DJ, Wijshake T, Conover CA, Campisi J, and van Deursen JM. (2016). Senescent intimal foam cells are deleterious at all stages of atherosclerosis. Science 354, 472–477. 10.1126/science.aaf6659. 27789842 PMC5112585

[219] Xu M, Palmer AK, Ding H, Weivoda MM, Pirtskhalava T, White TA, Sepe A, Johnson KO, Stout MB, Giorgadze N, (2015). Targeting senescent cells enhances adipogenesis and metabolic function in old age. Elife 4, e12997. 10.7554/eLife.12997. 26687007 PMC4758946

[220] Baker DJ, Wijshake T, Tchkonia T, LeBrasseur NK, Childs BG, van de Sluis B, Kirkland JL, and van Deursen JM. (2011). Clearance of p16Ink4a-positive senescent cells delays ageing-associated disorders. Nature 479, 232–236. 10.1038/nature10600. 22048312 PMC3468323

[221] Baker DJ, Childs BG, Durik M, Wijers ME, Sieben CJ, Zhong J, Saltness RA, Jeganathan KB, Verzosa GC, Pezeshki A, (2016). Naturally occurring p16(Ink4a)-positive cells shorten healthy lifespan. Nature 530, 184–189. 10.1038/nature16932. 26840489 PMC4845101

[222] Yamakoshi K, Takahashi A, Hirota F, Nakayama R, Ishimaru N, Kubo Y, Mann DJ, Ohmura M, Hirao A, Saya H, (2009). Real-time in vivo imaging of p16Ink4a reveals cross talk with p53. J Cell Biol 186, 393–407. 10.1083/jcb.200904105. 19667129 PMC2728398

[223] Liu JY, Souroullas GP, Diekman BO, Krishnamurthy J, Hall BM, Sorrentino JA, Parker JS, Sessions GA, Gudkov AV, and Sharpless NE. (2019). Cells exhibiting strong p16(INK4a) promoter activation in vivo display features of senescence. Proc Natl Acad Sci U S A 116, 2603–2611. 10.1073/pnas.1818313116. 30683717 PMC6377452

[224] Omori S, Wang TW, Johmura Y, Kanai T, Nakano Y, Kido T, Susaki EA, Nakajima T, Shichino S, Ueha S, (2020). Generation of a p16 Reporter Mouse and Its Use to Characterize and Target p16(high) Cells In Vivo. Cell Metab 32, 814–828.e816. 10.1016/j.cmet.2020.09.006. 32949498

[225] Chandra A, Lagnado AB, Farr JN, Doolittle M, Tchkonia T, Kirkland JL, LeBrasseur NK, Robbins PD, Niedernhofer LJ, Ikeno Y, (2022). Targeted clearance of p21- but not p16-positive senescent cells prevents radiation-induced osteoporosis and increased marrow adiposity. Aging Cell 21, e13602. 10.1111/acel.13602. 35363946 PMC9124310

[226] Hashimoto M, Asai A, Kawagishi H, Mikawa R, Iwashita Y, Kanayama K, Sugimoto K, Sato T, Maruyama M, and Sugimoto M. (2016). Elimination of p19(ARF)-expressing cells enhances pulmonary function in mice. JCI Insight 1, e87732. 10.1172/jci.insight.87732. 27699227 PMC5033852

[227] Jeck WR, Siebold AP, and Sharpless NE. (2012). Review: a meta-analysis of GWAS and age-associated diseases. Aging Cell 11, 727–731. 10.1111/j.1474-9726.2012.00871.x. 22888763 PMC3444649

[228] Farooq U, and Notani D. (2022). Transcriptional regulation of INK4/ARF locus by cis and trans mechanisms. Front Cell Dev Biol 10, 948351. 10.3389/fcell.2022.948351. 36158211 PMC9500187

[229] Toussaint O, Royer V, Salmon M, and Remacle J. (2002). Stress-induced premature senescence and tissue ageing. Biochem Pharmacol 64, 1007–1009. 10.1016/s0006-2952(02)01170-x. 12213599

[230] Robles SJ, Buehler PW, Negrusz A, and Adami GR. (1999). Permanent cell cycle arrest in asynchronously proliferating normal human fibroblasts treated with doxorubicin or etoposide but not camptothecin. Biochem Pharmacol 58, 675–685. 10.1016/s0006-2952(99)00127-6. 10413306

[231] Papadopoulou A, and Kletsas D. (2011). Human lung fibroblasts prematurely senescent after exposure to ionizing radiation enhance the growth of malignant lung epithelial cells in vitro and in vivo. Int J Oncol 39, 989–999. 10.3892/ijo.2011.1132. 21814715

[232] Ness KK, Kirkland JL, Gramatges MM, Wang Z, Kundu M, McCastlain K, Li-Harms X, Zhang J, Tchkonia T, Pluijm SMF, and Armstrong GT. (2018). Premature Physiologic Aging as a Paradigm for Understanding Increased Risk of Adverse Health Across the Lifespan of Survivors of Childhood Cancer. J Clin Oncol 36, 2206–2215. 10.1200/jco.2017.76.7467. 29874132 PMC6553838

[233] Ye J, Baer JM, Faget DV, Morikis VA, Ren Q, Melam A, Delgado AP, Luo X, Bagchi SM, Belle JI, (2024). Senescent CAFs Mediate Immunosuppression and Drive Breast Cancer Progression. Cancer Discov 14, 1302–1323. 10.1158/2159-8290.Cd-23-0426. 38683161 PMC11216870

[234] Belle JI, Sen D, Baer JM, Liu X, Lander VE, Ye J, Sells BE, Knolhoff BL, Faiz A, Kang LI, (2024). Senescence Defines a Distinct Subset of Myofibroblasts That Orchestrates Immunosuppression in Pancreatic Cancer. Cancer Discov 14, 1324–1355. 10.1158/2159-8290.Cd-23-0428. 38683144 PMC12155422

[235] Assouline B, Kahn R, Hodali L, Condiotti R, Engel Y, Elyada E, Mordechai-Heyn T, Pitarresi JR, Atias D, Steinberg E, (2024). Senescent cancer-associated fibroblasts in pancreatic adenocarcinoma restrict CD8(+) T cell activation and limit responsiveness to immunotherapy in mice. Nat Commun 15, 6162. 10.1038/s41467-024-50441-7. 39039076 PMC11263607

[236] Lee JY, Reyes N, Woo SH, Allen NC, Kadota T, Lechner A, Biswas R, Goel S, Stratton F, Kuang C, (2025). Senolytic-sensitive p16(Ink4a)+ fibroblasts in the tumor stroma rewire lung cancer metabolism and plasticity. Cell Stem Cell. 10.1016/j.stem.2025.10.005. PMC13186318 41187746

[237] Delire B, Henriet P, Lemoine P, Leclercq IA, and Starkel P. (2018). Chronic liver injury promotes hepatocarcinoma cell seeding and growth, associated with infiltration by macrophages. Cancer Sci 109, 2141–2152. 10.1111/cas.13628. 29727510 PMC6029836

[238] Luedde T, Beraza N, Kotsikoris V, van Loo G, Nenci A, De Vos R, Roskams T, Trautwein C, and Pasparakis M. (2007). Deletion of NEMO/IKKgamma in liver parenchymal cells causes steatohepatitis and hepatocellular carcinoma. Cancer Cell 11, 119–132. 10.1016/j.ccr.2006.12.016. 17292824

[239] Fane M, and Weeraratna AT. (2020). How the ageing microenvironment influences tumour progression. Nat Rev Cancer 20, 89–106. 10.1038/s41568-019-0222-9. 31836838 PMC7377404

[240] Rodier F, Coppé JP, Patil CK, Hoeijmakers WA, Muñoz DP, Raza SR, Freund A, Campeau E, Davalos AR, and Campisi J. (2009). Persistent DNA damage signalling triggers senescence-associated inflammatory cytokine secretion. Nat Cell Biol 11, 973–979. 10.1038/ncb1909. 19597488 PMC2743561

[241] Schmitt CA, Wang B, and Demaria M. (2022). Senescence and cancer - role and therapeutic opportunities. Nat Rev Clin Oncol 19, 619–636. 10.1038/s41571-022-00668-4. 36045302 PMC9428886

[242] Chaib S, Tchkonia T, and Kirkland JL. (2022). Cellular senescence and senolytics: the path to the clinic. Nat Med 28, 1556–1568. 10.1038/s41591-022-01923-y. 35953721 PMC9599677

[243] Zhu Y, Tchkonia T, Pirtskhalava T, Gower AC, Ding H, Giorgadze N, Palmer AK, Ikeno Y, Hubbard GB, Lenburg M, (2015). The Achilles' heel of senescent cells: from transcriptome to senolytic drugs. Aging Cell 14, 644–658. 10.1111/acel.12344. 25754370 PMC4531078

[244] Zhu Y, Tchkonia T, Fuhrmann-Stroissnigg H, Dai HM, Ling YY, Stout MB, Pirtskhalava T, Giorgadze N, Johnson KO, Giles CB, (2016). Identification of a novel senolytic agent, navitoclax, targeting the Bcl-2 family of anti-apoptotic factors. Aging Cell 15, 428–435. 10.1111/acel.12445. 26711051 PMC4854923

[245] Fuhrmann-Stroissnigg H, Niedernhofer LJ, and Robbins PD. (2018). Hsp90 inhibitors as senolytic drugs to extend healthy aging. Cell Cycle 17, 1048–1055. 10.1080/15384101.2018.1475828. 29886783 PMC6110594

[246] Triana-Martínez F, Picallos-Rabina P, Da Silva-Álvarez S, Pietrocola F, Llanos S, Rodilla V, Soprano E, Pedrosa P, Ferreirós A, Barradas M, (2019). Identification and characterization of Cardiac Glycosides as senolytic compounds. Nat Commun 10, 4731. 10.1038/s41467-019-12888-x. 31636264 PMC6803708

[247] Zhu Y, Doornebal EJ, Pirtskhalava T, Giorgadze N, Wentworth M, Fuhrmann-Stroissnigg H, Niedernhofer LJ, Robbins PD, Tchkonia T, and Kirkland JL. (2017). New agents that target senescent cells: the flavone, fisetin, and the BCL-X(L) inhibitors, A1331852 and A1155463. Aging (Albany NY) 9, 955–963. 10.18632/aging.101202. 28273655 PMC5391241

[248] Yousefzadeh MJ, Zhu Y, McGowan SJ, Angelini L, Fuhrmann-Stroissnigg H, Xu M, Ling YY, Melos KI, Pirtskhalava T, Inman CL, (2018). Fisetin is a senotherapeutic that extends health and lifespan. EBioMedicine 36, 18–28. 10.1016/j.ebiom.2018.09.015. 30279143 PMC6197652

[249] Xu M, Pirtskhalava T, Farr JN, Weigand BM, Palmer AK, Weivoda MM, Inman CL, Ogrodnik MB, Hachfeld CM, Fraser DG, (2018). Senolytics improve physical function and increase lifespan in old age. Nat Med 24, 1246–1256. 10.1038/s41591-018-0092-9. 29988130 PMC6082705

[250] Novais EJ, Tran VA, Johnston SN, Darris KR, Roupas AJ, Sessions GA, Shapiro IM, Diekman BO, and Risbud MV. (2021). Long-term treatment with senolytic drugs Dasatinib and Quercetin ameliorates age-dependent intervertebral disc degeneration in mice. Nat Commun 12, 5213. 10.1038/s41467-021-25453-2. 34480023 PMC8417260

[251] Ding N, Hah N, Yu RT, Sherman MH, Benner C, Leblanc M, He M, Liddle C, Downes M, and Evans RM. (2015). BRD4 is a novel therapeutic target for liver fibrosis. Proc Natl Acad Sci U S A 112, 15713–15718. 10.1073/pnas.1522163112. 26644586 PMC4697417

[252] Sanders YY, Lyv X, Zhou QJ, Xiang Z, Stanford D, Bodduluri S, Rowe SM, and Thannickal VJ. (2020). Brd4-p300 inhibition downregulates Nox4 and accelerates lung fibrosis resolution in aged mice. JCI Insight 5. 10.1172/jci.insight.137127. PMC7453889 32544088

[253] Amorim S, Stathis A, Gleeson M, Iyengar S, Magarotto V, Leleu X, Morschhauser F, Karlin L, Broussais F, Rezai K, (2016). Bromodomain inhibitor OTX015 in patients with lymphoma or multiple myeloma: a dose-escalation, open-label, pharmacokinetic, phase 1 study. Lancet Haematol 3, e196–204. 10.1016/s2352-3026(16)00021-1. 27063978

[254] Lewin J, Soria JC, Stathis A, Delord JP, Peters S, Awada A, Aftimos PG, Bekradda M, Rezai K, Zeng Z, (2018). Phase Ib Trial With Birabresib, a Small-Molecule Inhibitor of Bromodomain and Extraterminal Proteins, in Patients With Selected Advanced Solid Tumors. J Clin Oncol 36, 3007–3014. 10.1200/jco.2018.78.2292. 29733771

[255] Shapiro GI, LoRusso P, Dowlati A, K TD, Jacobson CA, Vaishampayan U, Weise A, Caimi PF, Eder JP, French CA, (2021). A Phase 1 study of RO6870810, a novel bromodomain and extra-terminal protein inhibitor, in patients with NUT carcinoma, other solid tumours, or diffuse large B-cell lymphoma. Br J Cancer 124, 744–753. 10.1038/s41416-020-01180-1. 33311588 PMC7884382

[256] Hamilton EP, Wang JS, Oza AM, Patel MR, Ulahannan SV, Bauer T, Karlix JL, Zeron-Medina J, Fabbri G, Marco-Casanova P, (2023). First-in-human Study of AZD5153, A Small-molecule Inhibitor of Bromodomain Protein 4, in Patients with Relapsed/Refractory Malignant Solid Tumors and Lymphoma. Mol Cancer Ther 22, 1154–1165. 10.1158/1535-7163.Mct-23-0065. 37486983 PMC10544002

[257] Xu M, Tchkonia T, Ding H, Ogrodnik M, Lubbers ER, Pirtskhalava T, White TA, Johnson KO, Stout MB, Mezera V, (2015). JAK inhibition alleviates the cellular senescence-associated secretory phenotype and frailty in old age. Proc Natl Acad Sci U S A 112, E6301–6310. 10.1073/pnas.1515386112. 26578790 PMC4655580

[258] Palmer AK, Xu M, Zhu Y, Pirtskhalava T, Weivoda MM, Hachfeld CM, Prata LG, van Dijk TH, Verkade E, Casaclang-Verzosa G, (2019). Targeting senescent cells alleviates obesity-induced metabolic dysfunction. Aging Cell 18, e12950. 10.1111/acel.12950. 30907060 PMC6516193

[259] Roos CM, Zhang B, Palmer AK, Ogrodnik MB, Pirtskhalava T, Thalji NM, Hagler M, Jurk D, Smith LA, Casaclang-Verzosa G, (2016). Chronic senolytic treatment alleviates established vasomotor dysfunction in aged or atherosclerotic mice. Aging Cell 15, 973–977. 10.1111/acel.12458. 26864908 PMC5013022

[260] Justice JN, Nambiar AM, Tchkonia T, LeBrasseur NK, Pascual R, Hashmi SK, Prata L, Masternak MM, Kritchevsky SB, Musi N, and Kirkland JL. (2019). Senolytics in idiopathic pulmonary fibrosis: Results from a first-in-human, open-label, pilot study. EBioMedicine 40, 554–563. 10.1016/j.ebiom.2018.12.052. 30616998 PMC6412088

[261] González-Gualda E, Pàez-Ribes M, Lozano-Torres B, Macias D, Wilson JR 3rd, González-López C, Ou HL, Mirón-Barroso S, Zhang Z, Lérida-Viso A, (2020). Galacto-conjugation of Navitoclax as an efficient strategy to increase senolytic specificity and reduce platelet toxicity. Aging Cell 19, e13142. 10.1111/acel.13142. 32233024 PMC7189993

[262] Amor C, Fernández-Maestre I, Chowdhury S, Ho YJ, Nadella S, Graham C, Carrasco SE, Nnuji-John E, Feucht J, Hinterleitner C, (2024). Prophylactic and long-lasting efficacy of senolytic CAR T cells against age-related metabolic dysfunction. Nat Aging 4, 336–349. 10.1038/s43587-023-00560-5. 38267706 PMC10950785

[263] Zhang Z, Ma B, Li B, Li Z, Gao M, Zhao H, Peng R, Hu J, Wang Y, You W, (2025). Cardiolipin-mimic lipid nanoparticles without antibody modification delivered senolytic in vivo CAR-T therapy for inflamm-aging. Cell Rep Med 6, 102209. 10.1016/j.xcrm.2025.102209. 40602406 PMC12281384

[264] Yang D, Sun B, Li S, Wei W, Liu X, Cui X, Zhang X, Liu N, Yan L, Deng Y, and Zhao X. (2023). NKG2D-CAR T cells eliminate senescent cells in aged mice and nonhuman primates. Sci Transl Med 15, eadd1951. 10.1126/scitranslmed.add1951. 37585504

[265] Lee S, and Schmitt CA. (2019). The dynamic nature of senescence in cancer. Nat Cell Biol 21, 94–101. 10.1038/s41556-018-0249-2. 30602768

[266] Däbritz JH, Yu Y, Milanovic M, Schönlein M, Rosenfeldt MT, Dörr JR, Kaufmann AM, Dörken B, and Schmitt CA. (2016). CD20-Targeting Immunotherapy Promotes Cellular Senescence in B-Cell Lymphoma. Mol Cancer Ther 15, 1074–1081. 10.1158/1535-7163.Mct-15-0627. 26880268

[267] Duro-Sánchez S, Nadal-Serrano M, Lalinde-Gutiérrez M, Arenas EJ, Bernadó Morales C, Morancho B, Escorihuela M, Pérez-Ramos S, Escrivá-de-Romaní S, Gandullo-Sánchez L, (2022). Therapy-Induced Senescence Enhances the Efficacy of HER2-Targeted Antibody-Drug Conjugates in Breast Cancer. Cancer Res 82, 4670–4679. 10.1158/0008-5472.Can-22-0787. 36222720 PMC9755966

[268] Wagner V, and Gil J. (2020). Senescence as a therapeutically relevant response to CDK4/6 inhibitors. Oncogene 39, 5165–5176. 10.1038/s41388-020-1354-9. 32541838 PMC7610384

[269] Eberlein CA, Stetson D, Markovets AA, Al-Kadhimi KJ, Lai Z, Fisher PR, Meador CB, Spitzler P, Ichihara E, Ross SJ, (2015). Acquired Resistance to the Mutant-Selective EGFR Inhibitor AZD9291 Is Associated with Increased Dependence on RAS Signaling in Preclinical Models. Cancer Res 75, 2489–2500. 10.1158/0008-5472.Can-14-3167. 25870145 PMC4605607

[270] Broderick C, Mezzadra R, Sisso EM, Mbuga F, Raghulan R, Chaves-Perez A, Kulick A, Jiang L, Jiang J, Ho YJ, (2025). A RAS(ON) Multi-Selective Inhibitor Combination Therapy Triggers Long-term Tumor Control through Senescence-Associated Tumor-Immune Equilibrium in Pancreatic Ductal Adenocarcinoma. Cancer Discov 15, 1717–1739. 10.1158/2159-8290.Cd-24-1425. 40299790 PMC12319406

[271] Wang L, Leite de Oliveira R, Wang C, Fernandes Neto JM, Mainardi S, Evers B, Lieftink C, Morris B, Jochems F, Willemsen L, (2017). High-Throughput Functional Genetic and Compound Screens Identify Targets for Senescence Induction in Cancer. Cell Rep 21, 773–783. 10.1016/j.celrep.2017.09.085. 29045843

[272] Troiani M, Colucci M, D'Ambrosio M, Guccini I, Pasquini E, Varesi A, Valdata A, Mosole S, Revandkar A, Attanasio G, (2022). Single-cell transcriptomics identifies Mcl-1 as a target for senolytic therapy in cancer. Nat Commun 13, 2177. 10.1038/s41467-022-29824-1. 35449130 PMC9023465

[273] Shahbandi A, Rao SG, Anderson AY, Frey WD, Olayiwola JO, Ungerleider NA, and Jackson JG. (2020). BH3 mimetics selectively eliminate chemotherapy-induced senescent cells and improve response in TP53 wild-type breast cancer. Cell Death Differ 27, 3097–3116. 10.1038/s41418-020-0564-6. 32457483 PMC7560696

[274] Kartika ID, Kotani H, Iida Y, Koyanagi A, Tanino R, and Harada M. (2021). Protective role of cytoplasmic p21Cip1/Waf1 in apoptosis of CDK4/6 inhibitor-induced senescence in breast cancer cells. Cancer Med 10, 8988–8999. 10.1002/cam4.4410. 34761877 PMC8683524

[275] Wang C, Vegna S, Jin H, Benedict B, Lieftink C, Ramirez C, de Oliveira RL, Morris B, Gadiot J, Wang W, (2019). Inducing and exploiting vulnerabilities for the treatment of liver cancer. Nature 574, 268–272. 10.1038/s41586-019-1607-3. 31578521 PMC6858884

[276] Wang TW, Johmura Y, Suzuki N, Omori S, Migita T, Yamaguchi K, Hatakeyama S, Yamazaki S, Shimizu E, Imoto S, (2022). Blocking PD-L1-PD-1 improves senescence surveillance and ageing phenotypes. Nature 611, 358–364. 10.1038/s41586-022-05388-4. 36323784

[277] Chaib S, López-Domínguez JA, Lalinde-Gutiérrez M, Prats N, Marin I, Boix O, García-Garijo A, Meyer K, Muñoz MI, Aguilera M, (2024). The efficacy of chemotherapy is limited by intratumoral senescent cells expressing PD-L2. Nat Cancer 5, 448–462. 10.1038/s43018-023-00712-x. 38267628 PMC10965441

[278] Forde PM, Spicer J, Lu S, Provencio M, Mitsudomi T, Awad MM, Felip E, Broderick SR, Brahmer JR, Swanson SJ, (2022). Neoadjuvant Nivolumab plus Chemotherapy in Resectable Lung Cancer. N Engl J Med 386, 1973–1985. 10.1056/NEJMoa2202170. 35403841 PMC9844511

[279] Altorki NK, Walsh ZH, Melms JC, Port JL, Lee BE, Nasar A, Spinelli C, Caprio L, Rogava M, Ho P, (2023). Neoadjuvant durvalumab plus radiation versus durvalumab alone in stages I-III non-small cell lung cancer: survival outcomes and molecular correlates of a randomized phase II trial. Nat Commun 14, 8435. 10.1038/s41467-023-44195-x. 38114518 PMC10730562

[280] Ngwa W, Irabor OC, Schoenfeld JD, Hesser J, Demaria S, and Formenti SC. (2018). Using immunotherapy to boost the abscopal effect. Nat Rev Cancer 18, 313–322. 10.1038/nrc.2018.6. 29449659 PMC5912991

[281] Liggett WH Jr., and Sidransky D. (1998). Role of the p16 tumor suppressor gene in cancer. J Clin Oncol 16, 1197–1206. 10.1200/jco.1998.16.3.1197. 9508208

[282] Abbas T, and Dutta A. (2009). p21 in cancer: intricate networks and multiple activities. Nat Rev Cancer 9, 400–414. 10.1038/nrc2657. 19440234 PMC2722839

[283] Gorgoulis V, Adams PD, Alimonti A, Bennett DC, Bischof O, Bishop C, Campisi J, Collado M, Evangelou K, Ferbeyre G, (2019). Cellular Senescence: Defining a Path Forward. Cell 179, 813–827. 10.1016/j.cell.2019.10.005. 31675495

